# Natural course of metabolically healthy phenotype and risk of developing Cardiometabolic diseases: a three years follow-up study

**DOI:** 10.1186/s12902-021-00754-1

**Published:** 2021-04-28

**Authors:** Daniel Elías-López, Arsenio Vargas-Vázquez, Roopa Mehta, Ivette Cruz Bautista, Fabiola Del Razo Olvera, Donaji Gómez-Velasco, Paloma Almeda Valdes, Carlos A. Aguilar-Salinas, for the Metabolic Syndrome Study Group, Olimpia Arellano-Campos, Donaji V. Gómez-Velasco, Omar Yaxmehen Bello-Chavolla, Ivette Cruz-Bautista, Marco A. Melgarejo-Hernandez, Paloma Almeda-Valdés, Alexandro J. Martagón, Liliana Muñoz-Hernandez, Luz E. Guillén, José de Jesús Garduño-García, Ulices Alvirde, Yukiko Ono-Yoshikawa, Ricardo Choza-Romero, Leobardo Sauque-Reyna, Ma. Eugenia Garay-Sevilla, Juan M. Malacara-Hernandez, María Teresa Tusié-Luna, Luis Miguel Gutierrez-Robledo, Francisco J. Gómez-Pérez, Rosalba Rojas, Carlos A. Aguilar-Salinas

**Affiliations:** 1 Unidad de Investigación de Enfermedades Metabólicas, Instituto Nacional de Ciencias Médicas y Nutrición Salvador Zubirán, Vasco de Quiroga 15. CP 14080; Tlalpan, México City, Mexico; 2 Departamento de Endocrinología y Metabolismo, Instituto Nacional de Ciencias Médicas y Nutrición Salvador Zubirán, México City, México; 3 MD/PhD (PECEM) Program, Faculty of Medicine, National Autonomous University of Mexico, Mexico City, Mexico; 4 Instituto Tecnologico y de Estudios Superiores de Monterrey Tec Salud, México City, México; 5 División de Nutrición, Instituto Nacional de Ciencias Médicas y Nutrición Salvador Zubirán, Mexico City, Mexico

**Keywords:** Obesity, Metabolically healthy obesity (MHO), Metabolically unhealthy obesity (MUHO), Cardiometabolic diseases, Metabolic health

## Abstract

**Background:**

Whether the metabolically healthy obese (MHO) phenotype is a single, stable or a transitional, fluctuating state is currently unknown. The Mexican-Mestizo population has a genetic predisposition for the development of type 2 diabetes (T2D) and other cardiometabolic complications. Little is known about the natural history of metabolic health in this population. The aim of this study was to analyze the transitions over time among individuals with different degrees of metabolic health and body mass index, and evaluate the incidence of cardiometabolic outcomes according to phenotype.

**Methods:**

The study population consisted of a metabolic syndrome cohort with at least 3 years of follow up. Participants were apparently-healthy urban Mexican adults ≥20 years with a body mass index (BMI) ≥20 kg/m2. Metabolically healthy phenotype was defined using the criteria of the National Cholesterol Education Program (*NCEP*) Adult Treatment Panel III (*ATP III*) metabolic syndrome criteria and the subjects were stratified into 4 groups according to their BMI and metabolic health. For cardiometabolic outcomes we estimated the incidence of cardiometabolic outcomes and standardized them per 1, 000 person-years of follow-up. Finally, to evaluate the risk for transition and development of cardiometabolic outcomes, we fitted Cox Proportional Hazard regression models.

**Results:**

Amongst the 5541 subjects, 54.2% were classified as metabolically healthy and 45.8% as unhealthy. The MHO prevalence was 39.3%. Up to a third of the population changed from their initial category to another and the higher transition rate was observed in MHO (42.9%). We also found several novel factors associated to transition to metabolically unhealthy phenotype; socioeconomic status, number of pregnancies, a high carbohydrate intake, history of obesity and consumption of sweetened beverages. Similarly, visceral adipose tissue (VAT) was a main predictor of transition; loss of VAT ≥5% was associated with reversion from metabolically unhealthy to metabolically healthy phenotype (hazard ratio (HR) 1.545, 95%CI 1.266–1.886). Finally, we observed higher incidence rates and risk of incident T2D and hypertension in the metabolically unhealthy obesity (MUHO) and metabolically unhealthy lean (MUHL) phenotypes compared to MHO.

**Conclusions:**

Metabolic health is a dynamic and continuous process, at high risk of transition to metabolically unhealthy phenotypes over time. It is imperative to establish effective processes in primary care to prevent such transitions.

**Supplementary Information:**

The online version contains supplementary material available at 10.1186/s12902-021-00754-1.

## Background

Obesity is a chronic and multifactorial disease associated with an increased risk for type 2 diabetes (T2D), arterial hypertension, atherogenic dislipidemia, nonalcoholic fatty liver disease, cardiovascular disease (CVD) and cancer [1–4]. Interestingly, a subgroup of overweight/obese patients remain free of cardiometabolic co-morbidities. This condition is known as the metabolically healthy obesity (MHO) phenotype. Currently, there is no universally accepted definition for these patients; up to 30 different criteria have been published [5–7]. As a consequence, the prevalence of the Metabolically healthy obesity phenotype varies enormously depending on the definition applied [8–10]. Several cohort studies have reported that the metabolically healthy obesity phenotype is not exempt for the development of incident T2D and cardiovascular disease [10–12]. However, the risk appears to be much lower than that reported for obese individuals with the metabolic syndrome (this phenotype is known as metabolically unhealthy obesity (MUHO)). Furthermore, different cohort studies with differing years of follow-up, have reported that between 30 and 50% of metabolically healthy obesity people develop metabolic complications over time [13, 14]. Some authors have proposed that MHO individuals are a unique group of obese patients resistant to the development of cardiometabolic complications. Thus, additional studies are needed to confirm if metabolically healthy obesity is a transition state or a stable phenotype [5]. In the Mexican population, little is known about the natural history of metabolically healthy obesity. In addition, the percentage of cases that transition between metabolically healthy (MH) phenotypes to metabolically unhealthy phenotypes (MUH) has not been reported for in Mexican Mestizos. The Mexican Mestizo population has raised over the last 500 years, of an admixture among Europeans (primarily Spaniards), Native-Americans, and Africans. Currently, they have formed the majority of the contemporary Mexican population from Mexico (~ 93%) [15]. This is a population with a genetic predisposition for the development of T2D and other cardiometabolic complications. Therefore, the objectives of the study are: To determine the prevalence of different BMI and metabolic health phenotypes in a Mexican population of apparently healthy adults; to analyze the transitions occurring in this cohort among individuals with different degrees of metabolic health (metabolically healthy and metabolically unhealthy) and body mass index (BMI) over a 3-year period; to determine the factors associated with transitions between phenotypes; and to inform about the incidence of cardiometabolic outcomes according to phenotype, during follow-up.

## Methods

### Study population

The study population was obtained from the Metabolic Syndrome (MS) cohort. This was a three-year study that evaluated MS components in people who developed incident type 2 diabetes, arterial hypertension and cardiovascular mortality in an urban population living in central Mexico (nine cities). The characteristics of the MS cohort have been described earlier [16]. Briefly, the study sample was composed of apparently-healthy adults ≥20 years with a BMI ≥20 kg/m2. At baseline, individuals with previously diagnosed T2D (self-reported) or a fasting plasma glucose ≥126 mg/dL confirmed in the first visit, coronary artery disease (CAD), cerebrovascular disease, alcoholism, taking corticosteroids, liver disease, chronic kidney disease or life-threatening diseases that would prevent the three-year follow-up, were excluded. Subjects were identified, invited, and evaluated on a voluntary basis, **through invitations to various workplaces** (offices of the federal government or private companies), homes, or during a visit of a relative to a medical unit. Invitations were made directly, through phone calls or e-mails. Patients had enough time to decide to participate in the study, all their doubts were resolved. Two evaluations were performed, baseline and final, in both the same procedures were performed, as described below.

### Clinical and anthropometric evaluations

All assessments were performed in the morning. The evaluation consisted of a clinical examination using standardized questionnaires (supplementary material), anthropometric measurements and a blood draw (a blood sample was obtained after a 9–12 h fast for the measurement of serum glucose, insulin, total cholesterol (TC) and high density lipoprotein-cholesterol (HDL-C), triglycerides (TG), apolipoprotein B (apo B), and C-reactive protein (CRP) levels). Weight was measured in kilograms and height in meters: the body mass index was calculated by dividing the weight by the height in meters squared. Waist to height ratio (WHtR) was calculated dividing the waist circumference (in centimeters) by the height (in centimeters). Participants were interviewed to obtain a medical history (including personal and family history of the most common chronic diseases), sociodemographic data, dietary information (24-h diet recall, 7-day food frequency questionnaire, the three-factor eating questionnaire) and physical activity habits (evaluated by short version of the International physical activity questionnaire (IPAQ) and defined as active or inactive) [17]. Two blood pressure measurements were obtained 10 min apart while participants were seated, and the average of the two measurements was used in the analysis. Personnel (physicians, nutritionists, nurses) were trained to administer and fill out the questionnaires. Anthropometric measurements were performed by trained and validated personnel.

Some of the variables were analyzed as continuous and/or categorical in the univariate analyses: glucose (< 90 or ≥ 90 mg/dL), age (< 50 or ≥ 50 years), apoB (>90th percentile for sex, < 99 or ≥ 99 mg/dL for women and < 108 or ≥ 108 mg/dL for men) and HDL-C (< 40 or ≥ 40 mg/dL for men and < 50 or ≥ 50 mg/dL for women). Consumption of sweetened beverages was defined as intake < 4 or ≥ 4 times a week, history of childhood obesity was defined as the diagnosis of obesity before the age of 18 and socio-economic status was defined as low, medium or high, as referred to by the subjects. All information collected was anonymized and kept confidential. The patients did not receive any incentive for taking part in the study.

Subjects were contacted after a three-year period (±6 months) and invited to repeat the evaluations using the same tools and methods. The response rate was 80.7%. The study was approved by the Ethics Committee of the Instituto Nacional de Ciencias Médicas y Nutrición and all participants signed an informed consent form. Patients were reconsented and informed about their voluntary participation in the second evaluation.

### Biochemical evaluations

All serum samples were kept frozen until processed in a central laboratory certified by the External Comparative Evaluation of Laboratories Program of the College of American Pathologists (Departamento de Endocrinología y Metabolismo, Instituto Nacional de Ciencias Médicas y Nutrición, México City). Plasma glucose concentration was measured by an automated glucose analyzer (Yellow Springs Instruments Co.), serum insulin concentration was measured by using a chemiluminescent immunoassay (Beckman Coulter Access 2). Lipid concentrations (TC, triglycerides (TG), and HDL cholesterol), apolipoprotein A (apo A), apo B, uric acid, creatinine and hepatic enzymes were measured using colorimetric assays (Unicel DxC 600 Synchron Clinical System Beckman Coulter). The Homeostasis Model Assessment 2-insulin resistance (HOMA2-IR) and sensitivity (HOMA2-S) were calculated using fasting glucose and insulin using the HOMA2 calculator released by the Diabetes Trials Unit, University of Oxford: HOMA Calculator. Visceral adipose tissue (VAT) was calculated using Metabolic Score for Visceral Fat (METS-VF) equation [18]**,** described previously by our group. This novel surrogate index estimates intra-abdominal adipose tissue incorporating a non-insulin-based IR index, the Metabolic Score for Insulin Resistance (METS-IR) [19], anthropometric measures of body-fat distribution (WtHr), genre, and age. METS-VF has better correlation and performance with visceral adipose tissue by dual-energy X-ray absorptiometry (VAT-DXA) compared to other surrogate VAT indexes.

### BMI/metabolic health phenotypes and outcomes definitions

Metabolic health was defined using the criteria of the National Cholesterol Education Program (*NCEP*) Adult Treatment Panel III (*ATP III*) metabolic syndrome criteria revised by the American Heart Association/National Heart, Lung, and Blood Institute (AHA/NHLBI) scientific statement [20]: systolic blood pressure ≥ 130 mmHg or diastolic blood pressure ≥ 85 mmHg; fasting plasma glucose concentration ≥ 100 mg/dL; HDL-C concentration < 40 mg/dL in men and < 50 mg/dL in women; fasting plasma TG concentration ≥ 150 mg/dL; or treatment with antihypertensive, lipid lowering, or glucose-lowering medications. Subjects had to meet ≤1 of the criteria (waist was excluded) to be metabolically healthy. To define metabolically unhealthy participants, at least ≥2 criteria had to be met. In addition, BMI was categorized as lean (BMI < 25 Kg/m^2^) or as overweight-obese (BMI ≥25 Kg/m^2^). In this way, we obtained 4 phenotypes: 1) metabolically healthy lean (MHL), 2) metabolically unhealthy lean (MUHL), 3) metabolically healthy overweight/obese (MHO) and 4) metabolically unhealthy overweight/obese (MUHO), respectively. These 4 phenotypes served as a basis for analyzing the transitions that occurred in the cohort after 3 years of follow-up, and at the end to analyze in what proportion these phenotypes remained/transitioned.

**Fig. 1:**
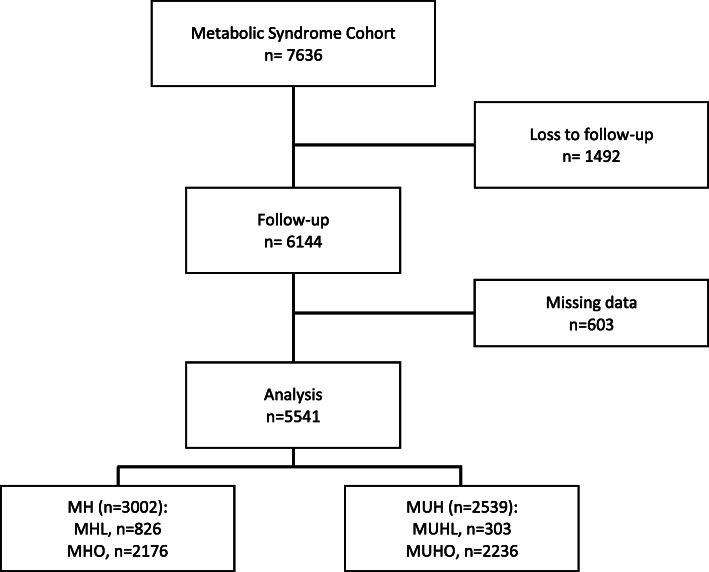
Flowchart of study participants at baseline and follow-up, outlining reasons for exclusion

At follow-up, incident T2D was defined as a previous diagnosis of type 2 diabetes, taking hypoglycemic medication and/or fasting glucose levels ≥7.0 mmol/dL (≥126 mg/dL) according to American Diabetes Association guidelines. Incident arterial hypertension was defined as previous medical diagnosis, taking antihypertensive medication and/or systolic blood pressure ≥ 140 mmHg or diastolic blood pressure ≥ 90 mmHg. Seven thousand six hundred thirty-six participants were recruited at baseline, of whom a total of 6144 participants agreed to continue with a follow-up visit. In total, 5541 had complete information to permit group classification, subjects those had missing data were excluded from the analysis (*n* = 603, 9.8%), Fig. 1.

### Statistical analysis

Data are presented as mean ± standard deviation (SD) or as median and interquartile range, categorical variables are expressed as frequencies and percentages. At baseline, clinical, biochemical and anthropometric variables were compared using the chi-square test and the Mann-Whitney-U test. To compare the clinical, biochemical, and anthropometric characteristics of the subjects during follow-up, we used the McNemar test for categorical variables and the Wilcoxon signed rank test or paired Student’s t-test according to the variable distribution (parametric or non-parametric) for continuous variables.

#### Risk factors associated to transitions between phenotypes

To evaluate factors associated to transitions between phenotypes, we divided the dataset according to the baseline phenotypes (metabolically healthy and unhealthy). For the metabolically healthy phenotype, the event was defined as subjects whose transitioned to unhealty phenotype and subjects whose remained as metabolically healthy were considered the reference group. In contrast, for the metabolically unhealthy phenotype, the event was defined as subjects whose reversed to healthy phenotype and subjects whose remained as metabolically unhealthy were considered the reference group. Then, we fitted Cox proportional hazard regression models to investigate the association of clinical, biochemical and anthropometric variables with the transitions between phenotypes, considering the transition or censoring in years, whichever comes first. The model assumptions were verified using Schöenfeld residuals. Univariate models and fully adjusted models were generated which included the following covariates: age, sex and BMI.

#### Cardiometabolic outcomes assessment

For cardiometabolic outcomes, we estimated the incidence rate for each outcome by dividing the number of new cases and standardized them per 1, 000 person-years of follow-up. Finally, to evaluate the risk for the development of cardiometabolic outcomes by phenotype, we fitted Cox Proportional Hazard regression models considering incident cardiometabolic outcome or censoring in years, whichever occurred first. Model assumptions were verified using Schöenfeld residuals. Adjusted models were generated which included the following covariates: age, sex, BMI, smoking, physical activity and family history of T2D and hypertension. A *p*-value < 0.05 was considered as the statistical significance threshold. All analyses were performed using R software version 3.6.2, Statistical Package for Social Sciences software (SPSS, version 25.0) and GraphPad Prism version 7.0.

## Results

### Baseline characteristics

Baseline characteristics are shown in (supplementary Table 1). Amongst the 5541 subjects, 3002 participants were classified as metabolically healthy (54.2%) and 2539 participants as metabolically unhealthy (45.8%). In the metabolically healthy phenotypes, subjects were younger, more often women, had a bachelor’s degree or higher and lower BMI compared to metabolically unhealthy phenotypes (*p* < 0.05).

According to BMI and metabolic health categories, subjects were classified as metabolically healthy lean (14.9%), metabolically unhealthy lean (5.4%), metabolically healthy overweight/obese (39.3%) and metabolically unhealthy overweight/obese (40.4%). Of note, 79.7% of the entire study population (*n* = 4412) had a BMI ≥25 Kg/m^2^. Metabolically healthy obese subjects had healthier habits in terms of smoking, alcohol consumption and physical activity compared to metabolically unhealthy obese subjects (*p* < 0.001). Moreover, metabolically healthy lean and metabolically healthy obese subjects had significantly higher HOMA2-S, but lower HOMA2-IR and VAT evaluated by METS-VF compared to metabolically unhealthy lean and metabolically unhealthy obese subjects (*p* < 0.001).

### Transitions between phenotypes

After 3 years of follow-up (±6 months), 63.4% of the subjects remained in the same group, while 36.6% of the individuals transitioned to another phenotype.

**Fig. 2:**
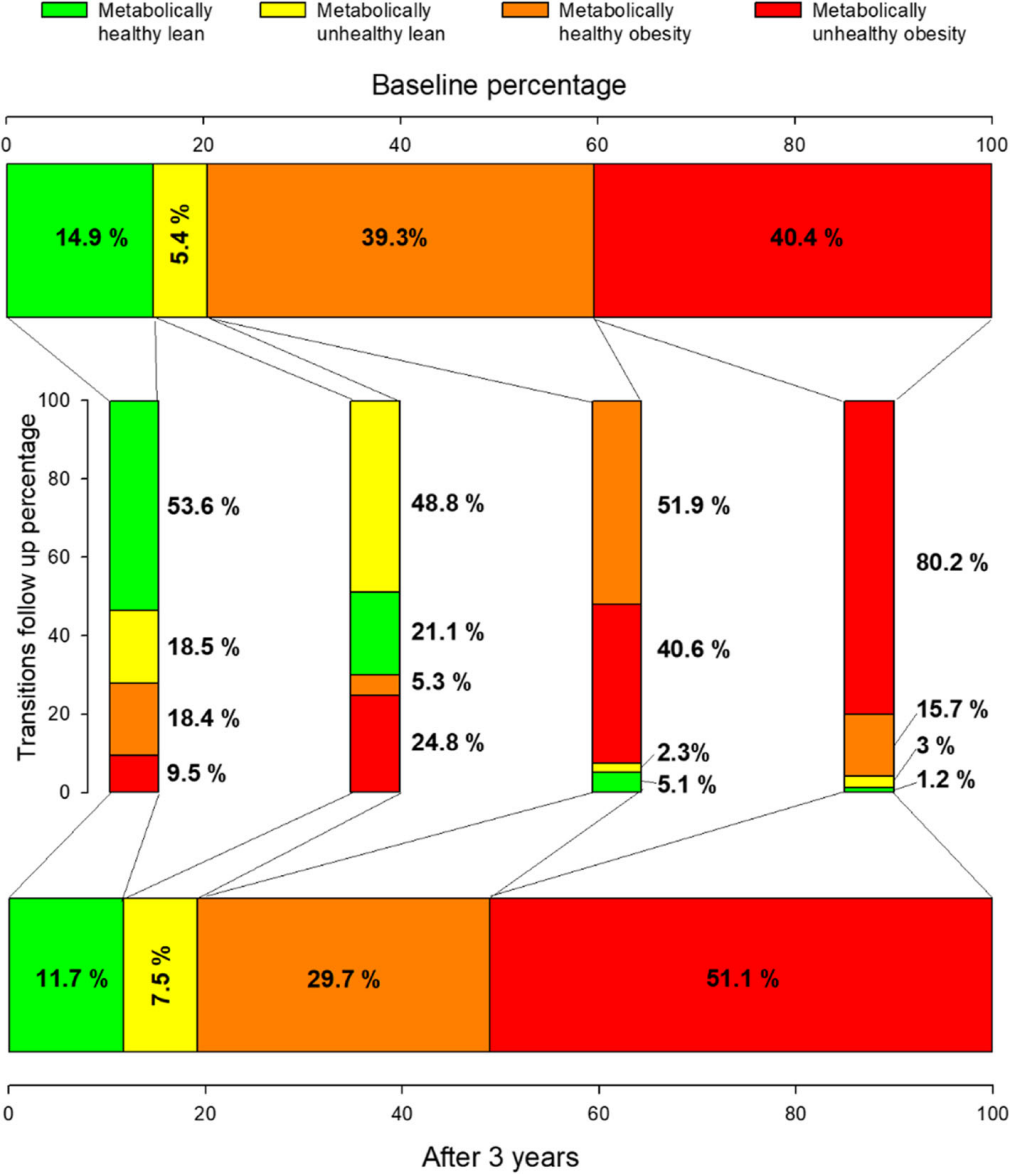
Permanence and transitions (progressions and reversals) of the 4 groups in the study

First, we analyzed the cohort from the perspective of metabolic health, independent of BMI, and we found that 39.2% of the individuals who were metabolically healthy at baseline transitioned to metabolically unhealthy phenotype; and 17.9% who were metabolically unhealthy at baseline reverted to metabolically healthy phenotype. Hence, at follow-up, subjects in the metabolically healthy group decreased in proportion to 41.2% (baseline 54.2%) and those in the metabolically unhealthy group increased in proportion to 58.8% (baseline 45.8%) **(**Fig. 2**).**

Then, we analyzed the 4 phenotypes stratified by BMI and metabolic health. We found that 53.6% (*n* = 443) of the metabolically healthy lean subjects remained in the same phenotype. However, 18.5% (*n* = 153) progressed to metabolically unhealthy lean, 18.4% (*n* = 152) to metabolically healthy obesity and 9.5% (*n* = 78) to metabolically unhealthy obesity (Fig. 2).

In the metabolically unhealthy lean group, 48.8% (*n* = 148) remained in the same phenotype, 5.2% (*n* = 16) transitioned to metabolically healthy obesity and 24.8%, (*n* = 75) to metabolically unhealthy obesity phenotypes, while 21.1% (*n* = 64) reverted to the metabolically healthy lean phenotype.

In the metabolically healthy obesity phenotype, 51.9% (*n* = 1130) remained in the same phenotype-. Interestingly, only 5.1% (*n* = 112) and 2.3% (*n* = 50) of individuals in this group reverted to metabolically healthy and unhealthy lean groups respectively, while the majority (40.6%, *n* = 884) progressed to metabolically unhealthy obesity phenotype.

Finally, in the metabolically unhealthy obesity group, 80.2% (*n* = 1793) remained in the same phenotype, while 1.2% (*n* = 27), 3% (*n* = 66) and 15.7% (*n* = 350) transitioned to metabolically healthy lean, metabolically unhealthy lean and metabolically healthy obese phenotypes, respectively.

Thus, at the time of follow-up the proportion of subjects in the 4 different phenotypes were as follow: 11.7% metabolically healthy lean (21.5% reduction), 7.5% metabolically unhealthy lean (37% increase), 29.7% metabolically healthy overweight/obese (24.25% reduction) and 51.1% metabolically unhealthy overweight/obese (26.5% increase) **(**Fig. 2**).**

### Factors associated with transitions between phenotypes

We analyzed parameters that allow us to identify which participants will progress to another phenotype. First, we compared basal characteristics from participants who transitioned with those who remained in the same group, according to metabolic health.

We found that subjects who progressed from metabolically healthy to metabolically unhealthy phenotype were older, more often men, had higher BMI, C-reactive protein, insulin and ApoB levels, (*p* < 0.001). Also, they had higher values of HOMA-IR, WHtR and METS-VF, *p* < 0.001 (Supplementary Table 2). The opposite was observed in those subjects who reverted from the metabolically unhealthy to the metabolically healthy profile. We fitted Cox-proportional hazard models to evaluate factors associated with the transitions. In the univariate analysis, progression from metabolically healthy to metabolically unhealthy phenotypes showed a positive association with age, gender (male), higher BMI, WHtR, METS-VF, HOMA2-IR, glucose and Apo B concentrations, a diet high in carbohydrates (> 60%) and C-reactive protein levels. (supplementary Table 3). Furthermore, subjects who remained maintained or gained weight had higher risk of progression to an unhealthy phenotype compared with subjects who lost weight. In contrast, the variables associated with an increased probability of reverting from a metabolically unhealthy phenotype to a metabolically healthy were greater HOMA-S and HDL-C levels and weight loss ≥5%.

Next, we evaluated the transition from metabolically healthy to metabolically unhealthy by BMI categories. The main predictors associated with a transition from metabolically healthy lean to metabolically unhealthy lean were gender (male), age, HOMA2-IR, METS-VF, WHtR, glucose, Apo B and number of pregnancies, whilst HDL-cholesterol and socioeconomic status were factors associated with a decreased risk of progression. For the metabolically healthy obese subjects, in addition to the aforementioned factors, BMI, history of childhood obesity, and a diet with > 60% carbohydrates intake were associated with higher risk of progression to metabolically unhealthy obesity; weight loss > 5%, physical activity, HDL-C, vegetables consumption (> 2 days per week) and HOMA2-S > 80% were associated with a lower risk of progression (Supplementary Table 3).

Factors associated with lower probability of reversion from the metabolically unhealthy lean category to metabolically healthy lean were gender (male), age, METS-VF and glucose. In contrast, HDL-cholesterol, weight loss ≥5% and HOMA2-S ≥ 80% were associated with a higher risk of reversion from metabolically unhealthy obesity to metabolically healthy obesity, whilst sugar sweetened beverage consumption and number of pregnancies were associated with a lower risk of regression. (Supplementary Table 4).

### Visceral adipose tissue as predictive factor to transitions between phenotypes

METS-VF (a VAT estimator) was associated with transitions between phenotypes. First, we evaluated the effect of VAT in the transitions between phenotypes according to BMI categories. We observed that metabolically healthy lean subjects with VAT ≥600 g had a higher risk of progression to a metabolically unhealthy lean phenotype, whilst metabolically healthy obese subjects with VAT ≥800 g had a higher risk of progression to a metabolically unhealthy obesity; [β = 0.441, HR 1.555, 95% confidence interval (CI) 1.139–2.121, *p* = 0.005] and [β = 0.279, HR 1.322, 95%CI 1.103–1.584, *p* = 0.002] respectively.

Subsequently, we analyzed the changes in VAT, and observed that loss of VAT ≥5% was associated with a 54.5% probability of reversion from metabolically unhealthy to metabolically healthy phenotype [β = 0.435, HR 1.545, 95%CI 1.266–1.886].

Finally, we evaluated the changes (%) in VAT according to changes in weight and we found that an increase in VAT in patients who lost weight (≥5%) or remained with stable weight were associated to lower risk of reversion from metabolically unhealthy to metabolically healthy phenotype; [β = − 0.014, HR 0.9857, 95%CI 0.996–1.000, *p* = 0.087] and [β = − 0.016, HR 0.983, 95%CI 0.974–0.992, *p* < 0.001] respectively. However, the changes in VAT were not associated to progression from metabolically healthy to metabolically unhealthy. All models were adjusted by age and BMI and stratified by sex.

### Metabolically healthy phenotype and cardiometabolic outcomes

**Fig. 3:**
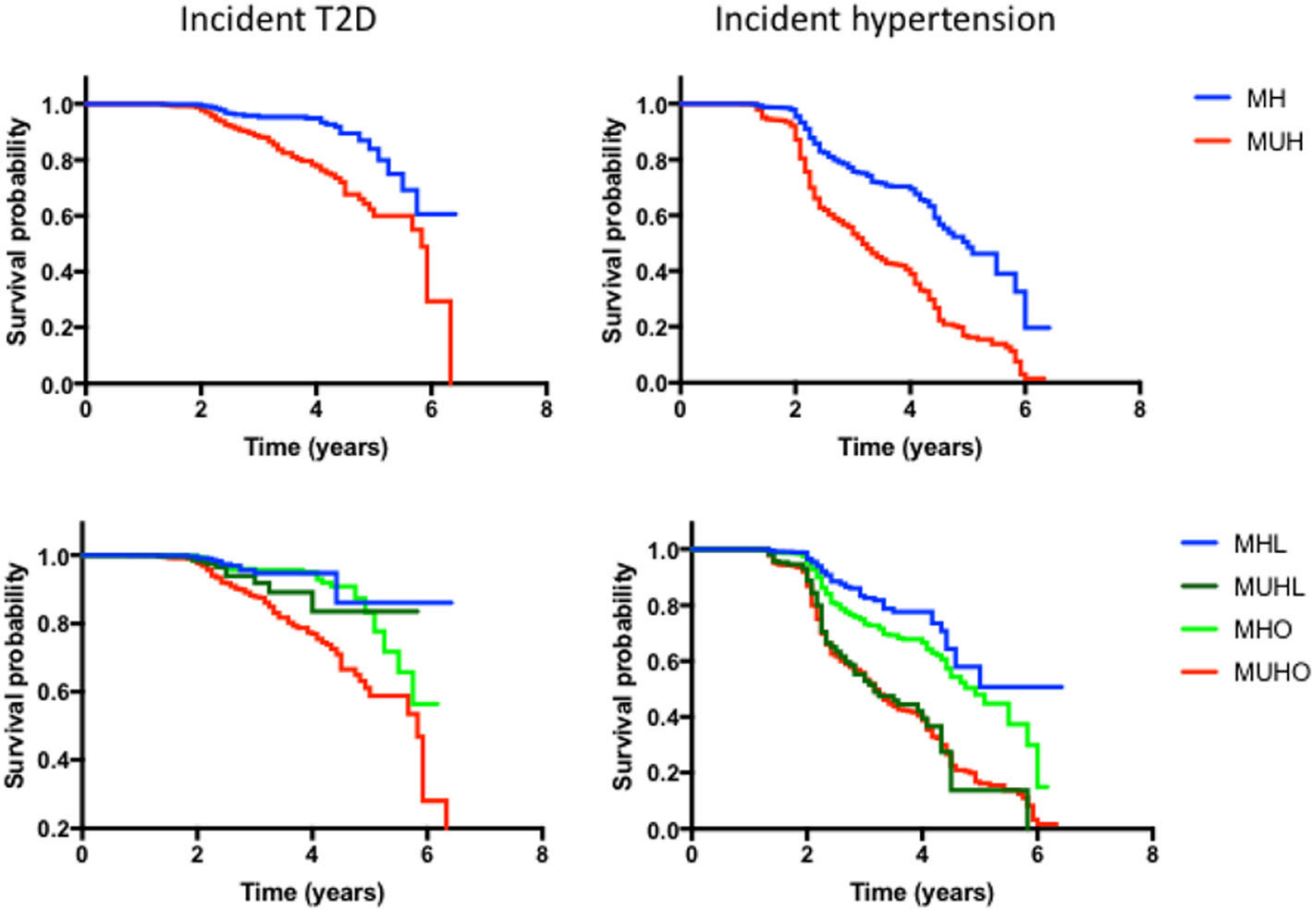
Kaplan-Meier survival curves for incident cardiometabolic outcomes according to stratified phenotype

We evaluated the cardiometabolic complications that developed in the cohort during follow-up. The groups were analyzed according to their metabolic health. In the metabolically healthy group, after 7215.41 person-years we identified 79 cases of incident T2D (incidence rate (IR) 10.94 per 1000 person-years, 95%CI 8.53–13.36), and 370 cases of incident hypertension (IR 51.28 per 1000 person-years, 95%CI 46.05–56.50). In contrast, after 6195.83 person-years, in the metabolically unhealthy subjects, there were 214 cases of incident T2D (IR 34.55 per 1000 person-years, 95%CI 29.92–39.18) and 447 cases of incident hypertension (IR 72.17 per 1000 person-years, 95%CI 65.48–78.86). It appears that the metabolically healthy phenotype is a protective factor for the development of incident T2D (HR 0.397, 95%CI 0.303–0.520) and hypertension (HR 0.807, 95%CI 0.698–0.933), (Fig. 3**)**. These models were adjusted for sex, BMI, age, smoking, physical activity and family history of T2D and hypertension.

Next, we evaluated the incidence rates according to BMI and metabolic health; here there was a lower incidence rate for any cardiometabolic outcome for the metabolically healthy lean phenotype (IR 9.62 CI 5.29–13.95 for diabetes and IR 36.98 CI 28.49–45.47 for hypertension) and the higher incidence for metabolically unhealthy obese phenotype (Table 1**)**. The metabolically healthy obese and metabolically unhealthy lean phenotypes had relatively similar incidence rates for hypertension; although for incident diabetes, there was a lower rate for metabolically healthy obese subjects compared to metabolically unhealthy lean.

**Table 1:** Cardiometabolic outcomes incidence rates (cases/1000 persons per year) in the study sample stratified by metabolic phenotype

Cardiometabolic outcome	Phenotype	Number of cases (%)	IR	95%CI
**Type 2 Diabetes**	**MHL**	**19 (2.3)**	**9.62**	**5.29–13.95**
	**MUHL**	**12 (4.0)**	**16.99**	**7.37–26.60**
	**MHO**	**60 (2.8)**	**11.44**	**8.55–14.34**
	**MUHO**	**202 (9.0)**	**36.81**	**31.73–41.49**
**Hypertension**	**MHL**	**73 (8.8)**	**36.98**	**28.49–45.47**
	**MUHL**	**39 (12.9)**	**55.22**	**37.88–72.55**
	**MHO**	**297 (13.6)**	**56.66**	**50.22–63.11**
	**MUHO**	**408 (18.2)**	**74.35**	**67.14–81.57**

Finally, we fitted Cox-proportional hazard models to evaluate the risk for the development of cardiometabolic outcomes by phenotype. We considered the metabolically healthy lean phenotype as a reference group (Fig. 3 and Table 2**)**. The metabolically unhealthy obese phenotype had the highest risk for incident T2D and hypertension (HR 2.21 95% CI 1.319–3.716, *p* = 0.003 and HR 2.355 95% CI 1.844–3.007 *p* = < 0.001 respectively); metabolically unhealthy lean showed increased risk for incident hypertension (HR 2.615 95% CI 1.319–3.716, *p* = 0.003) but not T2D. MHO subjects were not at risk for diabetes and hypertension in these models.

**Table 2:** Cardiometabolic complications developed by subjects during the following (divided by BMI and metabolic health). Models were adjusted by sex, BMI, age, smoking, physical activity and family history of T2D and hypertension

Cardiometabolic outcome	Phenotype	Beta	Wald	HR	95CI%	***p***
**Type 2 Diabetes**	**MHL**	**–**	**–**	**–**	**–**	**–**
	**MUHL**	**0.539**	**2.086**	**1.715**	**0.825–3.564**	**0.149**
	**MHO**	**−0.188**	**0.459**	**0.829**	**0.481–1.427**	**0.498**
	**MUHO**	**0.795**	**9.044**	**2.214**	**1.319–3.716**	**0.003**
**Hypertension**	**MHL**	**–**	**–**	**–**	**–**	**–**
	**MUHL**	**0.961**	**40.583**	**2.615**	**1.946–3.515**	**< 0.001**
	**MHO**	**0.192**	**2.277**	**1.211**	**0.944–1.554**	**0.131**
	**MUHO**	**0.857**	**47.127**	**2.355**	**1.844–3.007**	**< 0.001**

## Discussion

This observational study describes the metabolic health transitions that occur over a 3-year time period in an unbiased set of Mexican adults. Despite being an apparently healthy population at baseline, only a fraction of the participants were metabolically healthy lean (14.9%) using the ATPIII criteria. In addition, almost 80% of the cohort had a BMI ≥25 Kg/m2, which coincides with the high prevalence of overweight/obesity in our country [21]. Different metabolically healthy obesity prevalences have been reported. In our population, the metabolically healthy obesity prevalence was 39.3%. In Europe, the prevalence of metabolically healthy obesity was 26%, Southeast Asia 37% and North America 43%, whilst higher prevalences haven been reported in South America and African populations, 71 and 86% respectively. In contrast, the lowest prevalence have been identified in the Middle East (23%) [22–24].

Our data suggest that metabolic health is a dynamic and continuous process. Up to a third of the population changed from their initial category to another. Most of the transitions occurred in the direction of the unhealthy phenotype. Several studies have reported such changes, however there are dramatic differences among studies, as the findings depend on the criteria used to define metabolic health, the BMI cut-off to define normal weight/obesity, the follow-up time, as well as the predominance of certain characteristics such as gender, age and race/ethnicity [25–35]. However, all the cohorts coincided with metabolic deterioration over time. In these cohorts, 33–52% of individuals metabolically healthy transitioned to a metabolically unhealthy phenotype. The Nurses’ Health Study [31], one of the studies with the longest follow-up (30 years), reported that healthy women transitioned to a metabolically unhealthy status over time in all BMI categories, although the risk was higher in those with a greater degree of adiposity compared to those at normal weight (84% vs 68%). Even though the follow-up of our cohort was relatively short, we documented that the higher transition rate was observed in metabolically healthy obese compared to metabolically healthy lean (42.9% vs 27.9% respectively). This evidence provides strong support for considering the metabolically healthy obesity condition as a transition stage rather a unique stable condition.

Several studies have examined the transitions between phenotypes [29–35]. Factors that have previously been described as predictors of transition to a metabolically unhealthy phenotype have also been documented in this study; in particular sociodemographic factors, adiposity and insulin resistance (age, gender, BMI, lipids, glucose, HOMA-IR, HOMA-S, VAT, WHtR, among others). However, this analysis shows that other factors may increase the risk of progressing to a metabolically unhealthy phenotype in our population, especially in subjects with a BMI ≥25 Kg/m2. These include socioeconomic status, number of pregnancies, a high carbohydrate diet, a low intake of vegetables, a low level of physical activity, history of obesity before the age of 18, and consumption of sweetened beverages (the latter was not statistically significant but showed a trend, with a *p* = 0.063). Conversely, factors that prevent the reversion from a metabolically unhealthy phenotype to a metabolically healthy profile, in those with BMI ≥25 Kg/m2 included smoking, alcohol consumption and consumption of sweetened beverages. Many of these lifestyle factors are prevalent in our population. According to data from the National Health and Nutrition Survey 2016, more than 80% of children and 35% of adolescents in Mexico do not meet the physical activity recommendations [36]. The prevalence of physical inactivity in adults was 29% in 2018 [21]. The percentage of adults who eat vegetables is less than 50% (44.7%) and among children and adolescents it is 22 and 24.9% respectively [21]. Our country has a high prevalence of overweight/obesity in children and adolescents (35.6 and 38.5% respectively). Alcohol consumption and smoking are found in 63.8 and 11.4% of adults respectively. Finally, the consumption of sweetened beverages is high, estimated at 85.9% in adults and similarly high in those under 18 years of age [21]. The prevalence of overweight and obese persons continues to grow, and the metabolic syndrome is frequent (between 36.8 and 49.8%, depending on the criteria used) [37].

Increased body fat is associated with the development of long-term cardiometabolic complications. However, fat distribution is a determining factor in the development of these complications, especially ectopic deposits such as visceral fat mass. Our results show that a higher amount of visceral fat is associated with progression towards a metabolically unhealthy phenotype, while those who have loss of visceral fat are more likely to revert to a metabolically healthy phenotype, even regardless of the BMI. Interestingly, we found that the increase of VAT even in subjects who lose weight is associated to higher risk of progression to unhealthy phenotype. Different studies have reported that the higher visceral fat mass contributes to the pathophysiology of cardiometabolic diseases through different mechanisms such as inflammation, greater release of free fatty acids in the circulation and alterations in the secretion of adipokines [38].

The incidence and risk of cardiometabolic diseases attributable to metabolic health have been analyzed in several studies [30–35, 39–41]. In the Framingham study [32], there was a higher risk for incident T2D and arterial hypertension in the metabolically healthy obese subjects compared to the metabolically healthy lean subjects, but a lower risk compared to the metabolically unhealthy obese phenotype. In the Multi-Ethnic Study of Atherosclerosis (MESA) [33, 34], similar results were reported, but on analysis of the impact of ethnicity, interestingly the Hispanic group had a higher risk of transition to a metabolically unhealthy phenotype and for development of cardiometabolic outcomes than Caucasians and African-Americans. Our findings confirm a higher incidence of cardiometabolic outcomes in metabolically unhealthy phenotypes. The metabolically unhealthy lean subjects had a higher risk of developing cardiometabolic complications during follow-up, even higher than MHO phenotype. Similar findings have been reported in other studies [42–44]. Prospective studies show that the risk of incident diabetes and CV disease is higher among MUHL than in MHO persons [44]. The metabolically unhealthy lean phenotype is often not included in prevention programs.

Our study has strengths and limitations. First, we analyzed transitions between metabolic health categories stratified by BMI and evaluated the incidence and risk for the development cardiometabolic outcomes in a population with high genetic and environmental predisposition. This is the first study that analyzes the behavior among persons with differing degrees of metabolic health in Mexico, this is the main strength. A potential limitation is the relatively short follow-up time compared to other studies; this may influence the robustness of the findings. Also, the inclusion of apparently-healthy adults could have influenced the incidence of type 2 diabetes and hypertension. However, we observed a high transitions rate between phenotypes, and this allowed us to evaluate the risk factors associated to transitions and determinate risk categories between subjects across BMI and metabolic health status. The loss to follow-up represented a relatively minor proportion (19.5%) which allowed for an adequate estimate of transitions and cardiometabolic outcomes incidence. For last, the exclusion of subjects with missing data (9.5%), with no significant differences comparing subjects who did and did not have missing data, did not impact on statistical power to observe differences between phenotypes, transitions and incidence of diabetes and hypertension.

## Conclusion

Metabolic health is an unstable phenotype at high risk of transition to metabolically unhealthy phenotypes over time. The greatest risk for transition was observed in the metabolically healthy obese subjects. It is imperative that we establish effective processes in primary care to contain such transitions [45].. Such strategies should be prioritized in people with obesity, regardless of their metabolic health, but also among those who are at normal weight but have evidence of impaired metabolic health.

## Supplementary Information

**Additional file 1: Table S1.** Baseline characteristics of groups according to BMI and metabolic health. Abbreviations; T2D: Type 2 diabetes: CVD; Cardiovascular disease: BMI; Body mass index. **Table S2.** Predictors to identify participants who progress/revert to a metabolically healthy/metabolically unhealthy phenotype. Abbreviations; T2D: Type 2 diabetes: CVD; Cardiovascular disease: BMI; Body mass index. **Table S3.** Univariate analysis to evaluate factors associated with the transitions according to metabolic health. Abbreviations: BMI; Body mass index: WHtR; Waist-height ratio. **Table S4.** Factors associated with progression according to metabolic health and BMI. Abbreviations: BMI; Body mass index: WHtR; Waist-height ratio. **Table S5.** Factors associated with reversion according to metabolic health and BMI. Abbreviations: BMI; Body mass index: WHtR; Waist-height ratio.

**Additional file 2:.** Questionnaire.

## Data Availability

Data is available from the corresponding author upon reasonable request.

## Abbreviations

MHO: Metabolically healthy obese
MHL: Metabolically healthy lean
MUHL: Metabolically unhealthy lean
MUHO: Metabolically unhealthy obese
T2D: Type 2 diabetes
MS: Metabolic syndrome
CVD: Cardiovascular disease
MH: Metabolically healthy
MUH: Metabolically unhealthy
MESA: Multi-ethnic study of atherosclerosis
HOMA-IR: Homeostatic model assessment-insulin resistance
HOMA-S: Homeostatic model assessment-sensitivity
VAT: Visceral adipose tissue
WHtR: Waist-to-height ratio
BMI: Body mass index
METS-VF: Metabolic score for visceral fat
