# Supplementary material for: Natural course of metabolically healthy phenotype and risk of developing Cardiometabolic diseases: a three years follow-up study
Source: BMC Endocr Disord. 2021 Apr 28;21:85. doi: 10.1186/s12902-021-00754-1 (PMC8080399; doi:10.1186/s12902-021-00754-1)
Supplement: Supplementary file 1 — Additional file 1: Table S1. Baseline characteristics of groups according to BMI and metabolic health. Abbreviations; T2D: Type 2 diabetes: CVD; Cardiovascular disease: BMI; Body mass index. Table S2. Predictors to identify participants who progress/revert to a metabolically healthy/metabolically unhealthy phenotype. Abbreviations; T2D: Type 2 diabetes: CVD; Cardiovascular disease: BMI; Body mass index. Table S3. Univariate analysis to evaluate factors associated with the transitions according to metabolic health. Abbreviations: BMI; Body mass index: WHtR; Waist-height ratio. Table S4. Factors associated with progression according to metabolic health and BMI. Abbreviations: BMI; Body mass index: WHtR; Waist-height ratio. Table S5. Factors associated with reversion according to metabolic health and BMI. Abbreviations: BMI; Body mass index: WHtR; Waist-height ratio. [file 12902_2021_754_MOESM1_ESM.docx]

**Supplementary material.**

| **Variable** | **MHL**  **n=826 (14.9%)** | **MUHL**  **n=303 (5.4%)** | **p** | **MHO**  **n=2176 (39.3%)** | **MUHO**  **n=2236 (40.4%)** | **p** |
| --- | --- | --- | --- | --- | --- | --- |
| Sex (female) | 644 (78.0) | 165 (54.5) | <0.001 | 1595 (73.3) | 1278 (57.2) | <0.001 |
| Age (years) | 38.0 (32.0-45.0) | 46.0 (36.0-55.0) | <0.001 | 40.0 (34.0-46.0) | 44.0 (37.0-52.0) | <0.001 |
| Familial history of T2D | 364 (44.1) | 142 (46.9) | 0.402 | 1149 (52.8) | 1191 (53.3) | 0.729 |
| Familial history of hypertension | 468 (57.1) | 166 (55.0) | 0.528 | 1222 (56.7) | 1302 (58.5) | 0.246 |
| Familial history of CVD | 260 (31.7) | 92 (30.5) | 0.690 | 604 (28.0) | 632.0 (28.4) | 0.804 |
| Familial history of obesity | 375 (45.7) | 118 (39.1) | 0.046 | 1197 (55.6) | 1244 (55.9) | 0.834 |
| Scholarship  (>=bachelor's degree) | 442 (53.5) | 129 (42.7) | 0.001 | 916 (42.1) | 871 (39.0) | 0.034 |
| Physical activity (IPAC) | 516 (62.5) | 193 (63.7) | 0.706 | 813 (37.4) | 721 (32.2) | <0.001 |
| Smoke | 307 (37.2) | 123 (40.6) | 0.293 | 811 (37.3) | 992 (44.4) | <0.001 |
| Alcohol consumption | 419 (51.2) | 302 (51.3-14.9) | 0.961 | 1055 (49.0) | 1214 (54.6) | <0.001 |
| BMI (Kg/m2) | 23.7 (22.9-24.4) | 23.9 (23.0-24.6) | 0.011 | 28.3 (26.6-30.8) | 29.8 (27.5-32.7) | <0.001 |
| Systolic bood preasure (mmHg) | 110.0  (100.0-114.0) | 119.3 (110.0-130.0) | <0.001 | 114.0 (110.0-119.3) | 120.0 (112.0-130.0) | <0.001 |
| Diastolic blood preasure (mmHg) | 75.5 (70.0-79.0) | 79.0 (70.0-84.0) | <0.001 | 75.5 (70.0-80.0) | 80.0 (75.5-89.3) | <0.001 |
| Waist circumference (cm) | 81.0 (76.5-85.4) | 85.0 (80.0-90.0) | <0.001 | 92.0 (86.4-98.0) | 97.0 (91.0-103.0) | <0.001 |
| Hip circumference (cm) | 105.1  (99.5-105.1) | 103.8 (97.0-105.1) | <0.001 | 105.0 (103.8-105.1) | 105.1 (103.8-105.1) | 0.578 |
| Waist-height ratio | 0.50 (0.48-0.53) | 0.52 (0.50-0.55) | <0.001 | 0.57 (0.54-0.61) | 0.60 (0.56-0.64) | <0.001 |
| Glucose (mg/dL) | 82.0 (75.0-87.0) | 85.0 (79.0-93.0) | <0.001 | 84.0 (77.0-90.0) | 89.0 (82.0-98.0) | <0.001 |
| Triglycerides (mg/dL) | 162.2  (148.3-172.3) | 168.3 (156.2-186.2) | <0.001 | 166.3 (152.3-178.2) | 176.2 (162.2-194.2) | <0.001 |
| Total cholesterol (mg/dL) | 199.5  (173.0-228.0) | 206.0 (178.0-223.0) | <0.001 | 202.0 (177.0-230.0) | 204.0 (181.0-233.0) | 0.026 |
| HDL-Cholesterol (mg/dL) | 50.0 (44.0-59.0) | 38.0 (34.0-47.0) | <0.001 | 47.0 (42.0-53.0) | 37.0 (33.0-43.0) | <0.001 |
| Non-HDL Cholesterol | 145.5  (123.5-174.0) | 164.0 (140.0-187.0) | <0.001 | 154.0 (1129.0-181.0) | 165.0 (144.0-191.0) | <0.001 |
| LDL-Cholesterol (mg/dL) | 123.0  (102.0-143.8) | 129.1 (112.0-137.0) | 0.036 | 125.0 (107.0-142.0) | 127.0 (114.0-135.0) | 0.131 |
| Apolipoprotein B (mg/dL) | 95.5 (80.1-113.0) | 110.0 (93.5-130) | <0.001 | 102.0 (85.8-121.0) | 114.0 (97.5-132.0) | <0.001 |
| Insulin (μU/L) | 6.7 (4.8-9.3) | 8.4 (5.8-11.6) | <0.001 | 9.7 (6.9-13.3) | 12.6 (9.0-18.3) | <0.001 |
| C-reactive protein (mg/dL) | 1.0 (0.6-2.0) | 1.4 (0.7-2.6) | <0.001 | 1.9 (1.0-4.0) | 2.5 (1.3-4.8) | <0.001 |
| METS-IR | 33.9 (31.8-36.1) | 38.8 (35.1-40.9) | <0.001 | 42.7 (39.1-47.2) | 49.5 (45.0-55.2) | <0.001 |
| HOMA-IR | 0.8 (0.6-1.2) | 1.1 (0.7-1.5) | <0.001 | 1.2 (0.9-1.7) | 1.6 (1.1-2.3) | <0.001 |
| HOMA-B (%) | 107.9  (82.6-135.4) | 109.0 (85.4-138.1) | 0.481 | 129.4 (99.8-164.9) | 135.3 (104.9-175.3) | <0.001 |
| HOMA-S (%) | 118.9  (85.5-164.3) | 93.9 (68.4-137.5) | <0.001 | 81.4 (59.2114.7) | 61.3 (42.1-87.2) | <0.001 |
| METS-VF (gr) | 1111.2  (990.0-1290.0) | 1441.2  (1232.9-1678.8) | <0.001 | 1364.2 (1199.5-1598.0) | 1653.5  (1438.2-1954.5) | <0.001 |

**Supplementary Table 1:** Baseline characteristics of groups according to BMI and metabolic health. Abbreviations; T2D: Type 2 diabetes: CVD; Cardiovascular disease: BMI; Body mass index.

| **Variable** | **MH**  **n=1828** | **MH to MUH**  **n=1174** | **p** | **MUH to MH**  **n=466** | **MUH**  **n=2073** | **p** |
| --- | --- | --- | --- | --- | --- | --- |
| Sex (female) | 1444 (79.0) | 795 (67.7) | <0.001 | 318 (68.2) | 1125 (54.3) | <0.001 |
| Age (years) | 39.0 (33.0-45.0) | 41.0 (35.0-48.0) |  | 42.0 (34.0-50.0) | 45.0 (37.0-52.0) |  |
| Familial history of T2D | 917 (50.2) | 596 (50.8) | 0.747 | 241 (51.7) | 1092 (52.7) | 0.708 |
| Familial history of hypertension | 1022 (56.5) | 668 (57.3) | 0.650 | 270 (57.9) | 1198 (58.1) | 0.959 |
| Familial history of CVD | 528 (29.2) | 336 (28.8) | 0.839 | 132 (28.3) | 592 (28.7) | 0.873 |
| Familial history of obesity | 952 (52.6) | 620 (53.2) | 0.752 | 258 (55.5) | 1104 (53.5) | 0.442 |
| Scholarship  (>=bachelor's degree) | 842 (46.1) | 516 (44.0) | 0.260 | 181 (38.8) | 819 (39.6) | 0.767 |
| Physical activity (IPAC) | 1155 (63.2) | 726 (61.8) | 0.458 | 311 (66.7) | 1397 (67.4) | 0.786 |
| Smoke | 662 (36.2) | 458 (39.0) | 0.122 | 183 (39.3) | 932 (45.0) | 0.025 |
| Alcohol consumption | 912 (50.4) | 562 (48.3) | 0.250 | 239 (51.3) | 1130 (54.8) | 0.166 |
| BMI (Kg/m2) | 26.6  (24.4-29.2) | 27.7  (25.5-30.7) | <0.001 | 28.2  (25.7-31.1) | 29.4 (26.8-32.6) | <0.001 |
| Systolic blood pressure (mmHg) | 114.0  (105.0-118.0) | 114.0 (110.0-119.3) | <0.001 | 119.3  (110.0-124.0) | 120.0  (114.0-130.0) | <0.001 |
| Diastolic blood pressure (mmHg) | 75.5  (70.0-78.9) | 76.0 (70.0-80.0) | <0.001 | 80.0  (70.0-85.3) | 80.0 (75.5-90.0) | <0.001 |
| Waist circumference (cm) | 88.0  (81.0-94.6) | 92.5 (85.0-98.0) | <0.001 | 93.0  (86.0-100.3) | 96.0 (89.8-103.0) | <0.001 |
| Hip circumference (cm) | 105.1  (103.8-110.1) | 105.1 (103.8-110.1) | 0.400 | 105.1  (103.8-110.1) | 105.1  (103.8-110.1) | 0.524 |
| Waist-height ratio | 0.55 +  (0.52-0.59) | 0.57 (0.53-0.62) | <0.001 | 0.57  (0.54-0.63) | 0.59 (0.56-0,64) | <0.001 |
| Glucose (mg/dL) | 82.0 (76.0-88.0) | 85.0 (78.0-91.0) | <0.001 | 86.0 (79.0-93.0) | 89.0 (82.0-99.0) | <0.001 |
| Triglycerides (mg/dL) | 162.3  (154.3-180.0) | 168.2 (154.3-180.2) | <0.001 | 170.2  (156.3-184.3) | 176.3  (162.2-195.2) | <0.001 |
| Total cholesterol (mg/dL) | 196.0  (171.0-226.0) | 208.0 (183.0-235.0) | <0.001 | 200.5  (176.0-231.0) | 204.0  (181.0-233.0) | 0.054 |
| HDL-Cholesterol (mg/dL) | 49.0  (43.0-57.0) | 46.0 (41.0-51.0) | <0.001 | 39.0  (35.0-47.3) | 37.0 (33.0-43.0) | <0.001 |
| Non-HDL Cholesterol | 145.0  (123.0-172.0) | 161.0 (137.0-186.0) | <0.001 | 159.5  (137.0-184.3) | 166.0  (145.0-192.0) | 0.019 |
| LDL-Cholesterol (mg/dL) | 123.0  (102.0-140.0) | 129.0 (113.0-145.0) | <0.001 | 124.2  (106.0-138.0) | 129.0  (116.0-135.0) | <0.001 |
| Apolipoprotein B (mg/dL) | 95.5  (80.9-113.0) | 109.0 (91.1-126.0) | <0.001 | 110.0  (92.9-127.3) | 114.0 (97.8-132.0) | <0.001 |
| Insulin (μU/L) | 8.2 (5.8-11.6) | 9.7 (6.9-13.7) | <0.001 | 11.2 (7.8-15.5) | 12.3 (8.7-18.0) | <0.001 |
| C-reactive protein (mg/dL) | 1.57 (0.80-3.27) | 1.85 (0.98-4.05) | <0.001 | 2.05 (1.06-4.0) | 2.34 (1.21-4.72) | 0.043 |
| METS-IR | 38.6  (34.9-43.6) | 42.4 (38.0-47.3) | <0.001 | 44.9  (40.6-50.7) | 49.2 (43.9-55.2) | <0.001 |
| HOMA-IR | 1.0 (0.7-1.4) | 1.2 (0.8-1.7) | <0.001 | 1.4 (1.0-2.0) | 1.6 (1.1-2.3) | <0.001 |
| HOMA-B (%) | 118.9  (92.7-154.7) | 127.9 (99.5-164.25) | <0.001 | 131.2  (102.0-173.6) | 131.0  (102.0-171.6) | 0.749 |
| HOMA-S (%) | 95.4  (68.2-136.5) | 81.2 (56.7-115.9) | <0.001 | 71.4  (50.1-101.8) | 62.6 (42.9-89.8) | <0.001 |
| METS-VF (gr) | 1236.8  (1090.5-1443.3) | 1396.6 (1216.2-1657.6) | <0.001 | 1468.6  (1280.2-1692.5) | 1668.7  (1445.0-1967.2) | <0.001 |

**Supplementary table 2:** Predictors to identify participants who progress/revert to a metabolically healthy/metabolically unhealthy phenotype. Abbreviations; T2D: Type 2 diabetes: CVD; Cardiovascular disease: BMI; Body mass index.

| Status | Variable | Beta | HR | 95%CI | p |
| --- | --- | --- | --- | --- | --- |
| **Progression**  **MH to MUH** | Sex (male) | 0.301 | 1.351 | 1.195-1.529 | <0.001 |
|  | BMI (Kg/m^2^) | 0.178 | 1.195 | 1.129-1.265 | <0.001 |
|  | Age (years) | 0.103 | 1.109 | 1.043-1.180 | <0.001 |
|  | METS-VF | 0.305 | 1.357 | 1.27—1.446 | <0.001 |
|  | HOMA-IR | 0.161 | 1.175 | 1.085-1.273 | <0.001 |
|  | HOMA-S (%) | -0.247 | 0.780 | 0.736-0.827 | <0.001 |
|  | C-reactive protein (mg/dL) | 0.097 | 1.102 | 1.040-1.168 | <0.001 |
|  | Stable weight/weight gain | 0.282 | 1.327 | 1.108-1.589 | <0.001 |
|  | WHtR | 0.103 | 1.109 | 1.043-1.180 | <0.001 |
|  | Glucose >90mg/dL | 0.194 | 1.214 | 1.070-1.378 | 0.003 |
|  | HDL-cholesterol (mg/dL) | -0.186 | 0.830 | 0.740-0.931 | 0.001 |
|  | ApoB >90 percentile | 0.505 | 1.657 | 1.473-1.863 | <0.001 |
|  | Diet (carbohydrates>60%) | 0.234 | 1.264 | 1.106-1.444 | <0.001 |
| **Regression**  **MUH to MH** | Sex (male) | -0.481 | 0.618 | 0.508-0.751 | <0.001 |
|  | BMI (Kg/m^2^) | -0.326 | 0.721 | 0.656-0.793 | <0.001 |
|  | Age (years) | -0.159 | 0.852 | 0.781-0.930 | <0.001 |
|  | Alcohol consumption | -0.215 | 0.806 | 0.671-0.967 | 0.020 |
|  | Smoke status | -0.250 | 0.778 | 0.646-0.938 | 0.008 |
|  | WHtR | -0.233 | 0.792 | 0.718-0.873 | <0.001 |
|  | METS-VF | -0.551 | 0.575 | 0.519-0.639 | <0.001 |
|  | HOMA2-S (%) | 0.209 | 1.232 | 1.121-1.354 | <0.001 |
|  | HOMA2-IR | -0.168 | 0.844 | 0.787-0.907 | <0.001 |
|  | C-reactive protein (mg/dL) | -0.130 | 0.877 | 0.795-0.968 | 0.009 |
|  | Glucose >90mg/dL | -0.567 | 0.567 | 0.470-0.685 | <0.001 |
|  | HDL-cholesterol (mg/dL) | 0.510 | 1.665 | 1-367-2.027 | <0.001 |
|  | Weight loss >5% | 0.259 | 1.296 | 1.020-1.646 | 0.033 |

**Supplementary table 3:** Univariate analysis to evaluate factors associated with the transitions according to metabolic health. Abbreviations: BMI; Body mass index: WHtR; Waist-height ratio.

| **Status** | **Basal phenotype** | **Variable** | **Beta** | **HR** | **95%CI** | **p** |
| --- | --- | --- | --- | --- | --- | --- |
| **Progression**  **MH to MUH** | **MHL** | Sex (male) | 0.537 | 1.711 | 1.308-2.239 | <0.001 |
|  |  | BMI (Kg/m^2^) | 0.194 | 1.214 | 0.905-1.627 | 0.196 |
|  |  | Age > 50 years | 0.009 | 1.009 | 0.887-1.147 | 0.897 |
|  |  | METS-VF | 0.302 | 1.353 | 1.173-1.559 | <0.001 |
|  |  | HOMA2-IR | 0.353 | 1.423 | 1.131-1.791 | 0.003 |
|  |  | HOMA2-S (%) | -0.353 | 0.703 | 0.604-0.810 | <0.001 |
|  |  | Stable weight/weight gain | 0.444 | 1.559 | 0.994-2.445 | 0.053 |
|  |  | WHtR | 0.542 | 1.720 | 1.235-2.397 | 0.001 |
|  |  | Glucose >90mg/dL | 0.373 | 1.452 | 1.079-1.954 | 0.014 |
|  |  | HDL-cholesterol (mg/dL) | -0.030 | 0.970 | 0.958-0.982 | <0.001 |
|  |  | ApoB >90 percentile | 0.389 | 1.476 | 1.143-1.905 | 0.003 |
|  |  | Socioeconomic status | -1.464 | 0.231 | 0.057-0.932 | 0.040 |
|  |  | Preeclampsia | 0.530 | 1.699 | 0.948-3.045 | 0.075 |
|  |  | Number of pregnancies | 0.204 | 1.226 | 1.097-1.371 | <0.001 |
|  |  | History of fetal macrosomia | 0.417 | 1.517 | 0.928-2.479 | 0.096 |
|  | **MHO** | Sex (male) | 0.214 | 1.238 | 1.077-1.423 | 0.003 |
|  |  | BMI (Kg/m^2^) | 0.114 | 1.121 | 1.031-1.219 | 0.007 |
|  |  | Age >50 years | 0.201 | 1.223 | 1.071-1.397 | 0.003 |
|  |  | METS-VF | 0.258 | 1.294 | 1.197-1.399 | <0.001 |
|  |  | HOMA2-IR | 0.078 | 1.081 | 1.002-1.167 | 0.045 |
|  |  | HOMA2-S (%) | -0.175 | 0.840 | 0.784-0.899 | <0.001 |
|  |  | Weight loss >5% | -0.216 | 0.770 | 0.631-0.940 | 0.010 |
|  |  | WHtR | 0.157 | 1.170 | 1.007-1.359 | 0.040 |
|  |  | HDL-cholesterol (mg/dL) | -0.174 | 0.841 | 0.739-0.956 | 0.008 |
|  |  | ApoB >90 percentile | 0.498 | 1.646 | 1.441-1.880 | <0.001 |
|  |  | Preeclampsia | 0.234 | 1.263 | 0.975-1.637 | 0.077 |
|  |  | Vegetables consumption (>2 days per week) | -0.492 | 0.611 | 0.514-0.727 | <0.001 |
|  |  | Number of pregnancies | 0.098 | 1.103 | 1.059-1.149 | 0.001 |
|  |  | Physical activity | -0.143 | 0.867 | 0.759-0.989 | 0.034 |
|  |  | Diet (>60% carbohydrates) | 0.259 | 1.296 | 1.114-1.507 | 0.001 |
|  |  | Childhood obesity (<18 years old) | 0.300 | 1.350 | 1.032-1.765 | 0.028 |
|  |  | Sugar sweetened beverage consumption | 0.158 | 1.171 | 0.991-1.384 | 0.063 |

**Supplementary table 4:** Factors associated with progression according to metabolic health and BMI. Abbreviations: BMI; Body mass index: WHtR; Waist-height ratio.

| **Status** | **Basal phenotype** | **Variable** | **Beta** | **HR** | **95%CI** | **p** |
| --- | --- | --- | --- | --- | --- | --- |
| **Reversion**  **MUH to MH** | **MUHL** | Sex (male) | -0.697 | 0.498 | 0.308-0.804 | 0.004 |
|  |  | Age >50 | -0.609 | 0.544 | 0.330-0.895 | 0.017 |
|  |  | METS-VF | -0.350 | 0.705 | 0.564-0.880 | 0.002 |
|  |  | Glucose >90mg/dL | -0.545 | 0.580 | 0.355-0.948 | 0.030 |
|  | **MUHO** | Sex (male) | -0.439 | 0.645 | 0.521-0.799 | <0.001 |
|  |  | BMI (Kg/m^2^) | -0.339 | 0.713 | 0.619-0.820 | <0.001 |
|  |  | Age >50 years | -0.245 | 0.782 | 0.639-0.958 | 0.017 |
|  |  | METS-VF | -0.558 | 0.572 | 0.505-0.648 | <0.001 |
|  |  | HOMA2-IR | -0.173 | 0.841 | 0.779-0.909 | 0.001 |
|  |  | HOMA2-S (%) | 0.194 | 1.214 | 1.089-1.353 | 0.001 |
|  |  | C-reactive protein (mg/dL) | -0.094 | 0.910 | 0.815-1.017 | 0.097 |
|  |  | Weight loss >5% | 0.277 | 1.319 | 1.020-1.705 | 0.035 |
|  |  | WHtR | -0.453 | 0.636 | 0.497-0.813 | <0.001 |
|  |  | Glucose >90mg/dL | -0.543 | 0.581 | 0.473-0.714 | 0.001 |
|  |  | HDL-cholesterol (mg/dL) | 0.565 | 1.759 | 1.417-2.184 | 0.001 |
|  |  | Alcohol consumption | -0.233 | 0.792 | 0.648-0.968 | 0.023 |
|  |  | Smoking status | -0.292 | 0.746 | 0.608-0.917 | 0.005 |
|  |  | Number of pregnancies | -0.069 | 0.934 | 0.870-1.001 | 0.054 |
|  |  | Sugar sweetened beverage consumption | -0.309 | 0.734 | 0.563-0.957 | 0.022 |

**Supplementary table 5:** Factors associated with reversion according to metabolic health and BMI. Abbreviations: BMI; Body mass index: WHtR; Waist-height ratio.
