# Supplementary material for: Natural course of metabolically healthy phenotype and risk of developing Cardiometabolic diseases: a three years follow-up study
Source: BMC Endocr Disord. 2021 Apr 28;21:85. doi: 10.1186/s12902-021-00754-1 (PMC8080399; doi:10.1186/s12902-021-00754-1)
Supplement: Supplementary file 2 — Additional file 2:. Questionnaire. [file 12902_2021_754_MOESM2_ESM.docx]

**CREATION OF A PROSPECTIVE COHORT REPRESENTATIVE OF THE URBAN ADULT POPULATION OF MEXICO**

**PARTICIPANT´S NAME AND ADDRESS**

NAME:______________________________________________________________________________________________________________________

_______________________________________________________________________________________________________________________________

(ADDRESS, INCLUDING STREET, HOUSE NUMBER, APARTMENT NUMBER, BUILDING, NEIGHBORHOOD, TOWNSHIP AND ZIP CODE)

PHONE NUMBER: _________________________________ E-MAIL________________________________________________________________

TWO CONTACT PEOPLE: (NAME, ADDRESS, INCLUDING STREET, HOUSE NUMBER, APARTMENT NUMBER, BUILDING, NEIGHBORHOOD, TOWNSHIP AND ZIP CODE, PHONE NUMBER AND E-MAIL)

1. ______________________________________________________________________________________________________________________
2. ______________________________________________________________________________________________________________________

**Date:______________________________________________________________________________**

**City:** __________________________________________________________________________

Interviewer: ___________________________________________________________

**1. Socio-demographic background**

**1.1 Is the patient alive? Yes**:____ (GO TO Q. 1.5)

**No**_____

**1.2 Cause of death**:_________________________________________________________________________

**1.3 Document proving the cause of death**:_________________________________________

_______________________________________________________________________________________________

**1.4 Date of death**:_________________________________________________________________________

**1.5 Socioeconomic status______**

1. Low 2. Middle 3. High

**1.6. Highest degree or level of education completed**:____

1. 1st grade 6. 6th grade 11. Never attended

2. 2nd grade 7. Middle school or technical program 12. Refuses to answer

3. 3rd grade 8. High school

4. 4th grade 9. Bachelor’s Degree

5. 5th grade 10. Master’s degree or higher

**1.6. Years of education:** ________

**1.8. Main occupation:** ___________________________________

**1.9 If female (under 50 years)**

**Number of pregnancies _________**

**Number of abortions _________ Number of deliveries _________**

**2. Family History:**

| 2.1 Have any of your parents, siblings, uncles, aunts or grandparents have or had diabetes? | Yes ………………........……….. 1  Who?____________________________  No.............................................. 2  Doesn´t know...................................... 9 | [____] |
| --- | --- | --- |
| 2.2 Have any of your parents, siblings, uncles, aunts or grandparents have or had obesity? | Yes ………………........……….. 1  Who?____________________________  No.............................................. 2  Doesn´t know...................................... 9 | [____] |
| 2.3 Have any of your parents, siblings, uncles, aunts or grandparents have or had high blood pressure? | Yes ………………........……….. 1  Who?____________________________  No.............................................. 2  Doesn´t know...................................... 9 | [____] |
| 2.4 Have any of your parents, siblings, uncles, aunts or grandparents have or had a heart attack? | Yes ………………........……….. 1  Who?____________________________  No.............................................. 2  Doesn´t know...................................... 9 | [____] |

**3. Diabetes**

| 3.1 **Have you ever been told by a doctor that you have diabetes or high blood sugar level?** | Yes.....................................................................1  Yes, during pregnancy (gestational diabetes).....3  No………......................................................... 2 | | [____]  GO TO Q.4 |
| --- | --- | --- | --- |
| 3.2 **When were you first diagnosed with diabetes or with high blood sugar level?** | Less than a month ……………... 00  Doesn´t know........................................ 99 | | **months** [___\|___]  **years**  [___\|___] |
| 3.3 **Have you been told to take prescribed medicine in order to control diabetes or lower blood sugar?** | Yes........................................... 1  No.......................................... 2 | | [____]  GO TO Q. 3.6 |
| 3.4 Are you currently taking pills or insulin in order to control blood sugar? | **Yes, Which one?**  Insulin........................................... 1  Pills................................................ 2  Both……………………………. 3  None........................................... 4 | | [____]  GO TO Q. 3.6  GO TO Q. 3.6 |
| 3.5 How many times and how frequently do you use insulin? | Daily……..…………………………… 1  Weekly…….………………….…….….2  Refuses to answer ...................................8  Doesn´t know ….........................................9 | | [___i___]  Times  [___]  **Frequency** |
| 3.6 **Are you currently undergoing other treatment in order to control your blood sugar?**  **YOU CAN CHOOSE MORE THAN ONE ANSWER** | **Yes, Which one?**  Diet.........................................................  Exercise.................................................  Homeopathic medicine......…….............  Herbal medicine.....................................  Hemotherapy...................……….….....  Other.............................……..............…  No……………………………………... | 01  02  03  04  05  77  07 | [___\|___]  [___\|___]  [___\|___]  [___\|___]  [___\|___]  [___\|___] |
| 3.7 **Because of diabetes, which of the following preventive measures had you used to avoid complications?**  **YOU CAN CHOOSE MORE THAN ONE ANSWER** | Ophthalmologic examination ………………… 1  Take a daily aspirin pill.…………………...….. 2  Foot examination.…………………………...…..3  Renal function test...……………………….……4  None............................................................…….5  Other (specify)__________________________7 | | [____]  [____]  [____] |
| 3.8 **Because of diabetes have you…** | **Yes No**  **had leg or foot ulcers**  **that take more than 4 weeks to heal?**...….1 2  **had an amputation?**.................................... 1 2  **had decreased eyesight?**............................. 1 2  **had retinal damage.**.................................... 1 2  **lost your sight?**............................................ 1 2  **had a dialysis?**............................................. 1 2  **had a stroke?**............................................... 1 2  **suffered from a diabetic coma?**................. 1 2  **lost feeling in any part of your body?**........1 2 | | [____]  [____]  [____]  [____]  [____]  [____]  [____]  [____]  [____] |

**4. Hypertension**

| 4.1 **Have you ever been told by a doctor that you had high blood pressure?** | Yes .......................................................... 1  No ........................................................ 2 | | [____] |
| --- | --- | --- | --- |
| **Interviewer: CONTINUE ONLY IF THE PARTICIPANT IS A WOMAN, OTHERWISE GO TO Q. 4.5** | | | |
| 4.2 **Were you pregnant when you were told you had high blood pressure?** | Yes .................................................................... 1  No ..................................................................... 2 | | [____] |
| 4.3 **Has a doctor or health professional ever diagnosed you with hypertension only during pregnancy? (preeclampsia)** | Yes .................................................................... 1  No ...................................................................... 2 | | [____] |
| 4.4 **Have you had a baby that weighed over 3.5 kilograms or 7.7 lbs. at birth?** | Yes ………………........………………………. 1  No....................................................................... 2  Doesn´t know......................................................... 9 | | [____] |
| 4.5 **How long ago did a doctor told you to had high blood pressure?** | I haven’t been diagnosed with hypertension........ 00  **(GO TO Q.5)**  Less than a month ................................................. 01  Doesn´t know............................................................ 99 | | **Months** [___\|___]  **Years** [___\|___] |
| 4.6 **Are you currently taking pills in order to control your blood pressure?** | Yes ....................................................................... 1  No ......................................................................... 2  Doesn´t know ........................................................... 9 | | [____]  GO TO Q. 4.8 |
| 4.7 **How long have you been taking these pills?** | Less than a month ......................................................00  Doesn´t know…………………………………….……99 | | **Months** [___\|___]  **Years** [___\|___] |
| 4.8 **Where do you go for your blood pressure check-ups?** | IMSS OPORTUNIDADES ....…………………......01  IMSS ........................................................................02  Secretaría de Salud....................................................03  SEGURO POPULAR (SSA)....................................04  DIF, CRUZ ROJA, INI ……....................................05  ISSSTE, ISSSTE ESTATAL ..................................06  MARINA/DEFENSA, PEMEX ..............................07  PARTICULAR ........................................................08  HOSPITAL CIVIL ..................................................09  NATIONAL INSTITUTES …................................10  OTHER ………………...........................................77  DOESN´T KNOW........................................................99 | | [___\|___] |
| 4.9 **Are you currently undergoing other treatment to control your high blood pressure?** | **If yes, which of the following?**  Diet ...........................................................................1  Exercise .................................................................2  Homeopathy.................................................……..3  Herbal remedies ............................................……4  Reduce sodium intake……....................................5  Other ..……………………………………...……7  No ........................…...........………….....……….6 | | [____]  [____]  [____]  [____]  [____]  [____] |
| 4.10 **How many times and how frequently did you or someone else checked your blood pressure?** | Weekly ................................................  Monthly ..............................................  Annually...................................................  Doesn´t know.........................................  Doesn’t do it……................................... | 1  2  3  8  4 | [____]  **Frequency**  [___\|___]  **Times** |

**5. Obesity**

| 5.1 **Has a doctor/ dietitian/ nutritionist ever told you that you are or were overweight?** | Yes………………………………..…………..1  No…………………………………………..…2  Doesn´t know………..…………………......…9 | [____]  GO TO Q. 6 |
| --- | --- | --- |
| 5.2 **How old were you when you were told you had obesity?** | Refuses to answer.............................................. 88  Doesn´t know ……………................................ 99 | [___i___]  **Age in years** |
| 5.3 **What has been your maximum weight** (without pregnancy)? | Refuses to answer.............................................. 88  Doesn´t know ……………................................ 99 | [___\|___]  **kilograms** |
| 5.4 **Did you follow any treatment in order to control your weight?** | Yes………………………………….…………....1  No……………………………………………..…2  Doesn´t know………………………………...…..9 | [____] |
| 5.5 **During the last year, have you lost or gained weight?** | **Yes**  Gained weight ……………………………………1  Lost weight ………………………………….........2  Answers 3 to 6  GO TO Q. 6  No weight change ………………………………...3  Gained or lost weight due to pregnancy……...…...4  Refuses to answer…..…………………..…………8  Doesn´t know…………………………-…..………9 | [____] |
| 5.6 **How many kilograms did you gain (or lost)?** | GO TO Q. 6  Gained weight | [___\|___]  **kilograms** |
| **CONTINUE IF YOU LOST MORE THAN 5 KILOGRAMS, IF YOU LOST LESS THAN 5 KILOGRAMS GO TO QUESTION 6** | | |
| 5.6 **Was this weight loss intentional?** | Yes……………………………………………..1  No ……………………………………………...2  Refuses to answer ………………….………….8  Doesn´t know…………….……….……………9 | [____]  GO TO Q. 6 |
| 5.7 **What did you do to lose weight?**  **YOU CAN CHOOSE MORE THAN ONE ANSWER** | Ate less food………………..……..………………….01  Ate low calorie foods……………………………..….02  Ate low fat foods …………………………………….03  Exercised…………………….....………………….....04  Skipped meals….……………………..……………...05  Ate “diet” products …………………………...……...06  Used a weight loss liquid diet formula (i.e. SlimFast) 07  Participated in a weight loss program………..……….08  Followed a special diet announced on a magazine, internet, television or recommended by friends or family............................................................................09  Followed a special diet prescribed by a doctor/ dietitian/ nutritionist…………………………….........................10  Took diet pills prescribed by a doctor ………………..11  Took other medicines, herbs, supplements, or homeopathic medicine not needing a prescription……12  Increased physical activity…………….……………...13  Drank lots of water………...…………………………14  Other (specify) _______________________________77  Doesn´t know………………………………………....88  Refuses to answer…….……………………………….99 | [___\|___]  [___\|___]  [___\|___]  [___\|___]  [___\|___]  [___\|___] |

6- Eating disorders

Carefully read the following sentences. If you agree with the sentence or if it’s true, place an “X” over the box that says True (T). If you disagree with the sentence or if it’s false, place an “X” over the box that says False (F). Make sure to honestly answer all questions.

|  | True | False |
| --- | --- | --- |
| 1. When I smell a sizzling steak or see a juicy piece of meat, I find it very difficult to keep from eating, even if I have just finished a meal | T | F |
| 1. I usually eat too much at social situations, like parties and picnics. | T | F |
| 1. I am usually so hungry that I eat more than three times a day. | T | F |
| 1. When I have eaten my calorie limit, I am usually good about not eating any more. | T | F |
| 1. Dieting is so hard for me because I get too hungry. | T | F |
| 1. I deliberately take small helpings as a means of controlling my weight. | T | F |
| 1. Sometimes things just taste so good that I keep on eating, even when I am no longer hungry. | T | F |
| 1. Since I am often hungry, I sometimes wish that while I am eating, an expert would tell me that I have had enough or that I can have something more to eat. | T | F |
| 1. When I feel anxious, I find myself eating. | T | F |
| 1. Life is too short to worry about dieting. | T | F |
| 1. Since my weight goes up and down, I have gone on reducing diets more than once. | T | F |
| 1. I often feel so hungry that I just have to eat something. | T | F |
| 1. When I am with someone who is overeating, I usually overeat too. | T | F |
| 1. I have a pretty good idea of the number of calories in common food. | T | F |
| 1. Sometimes when I start eating, I just can’t stop. | T | F |
| 1. It is not difficult for me to leave something on my plate. | T | F |
| 1. At certain times of the day, I get hungry because I have gotten used to eating then. | T | F |
| 1. While on a diet, if I eat food that is not allowed, I consciously eat less for a period of time to make up for it. | T | F |
| 1. Being with someone who is eating often makes me hungry enough to also eat. | T | F |
| 1. When I feel blue, I often overeat. | T | F |
| 1. I enjoy eating too much to spoil it by counting calories or watching my weight. | T | F |
| 1. When I see a real delicacy, I often get so hungry that I have to eat right away. | T | F |
| 1. I often stop eating when I am not really full as a conscious means of limiting the amount that I eat. | T | F |
| 1. I get so hungry that my stomach often seems like a bottomless pit. | T | F |
| 1. My weight has hardly changed at all in the last ten years. | T | F |
| 1. I am always hungry, so it is hard for me to stop eating before I finish the food on my plate. | T | F |
| 1. When I feel lonely, I console myself by eating. | T | F |
| 1. I consciously hold back at meals in order not to gain weight. | T | F |
| 1. I sometimes get very hungry late in the evening or at night. | T | F |
| 1. I eat anything I want, any time I want. | T | F |
| 1. Without even thinking about it, I take a long time to eat. | T | F |
| 1. I count calories as a conscious means of controlling my weight. | T | F |
| 1. I do not eat some foods because they make me fat. | T | F |
| 1. I am always hungry enough to eat at any time. | T | F |
| 1. I pay a great deal of attention to changes in my figure. | T | F |
| 1. While on a diet, if I eat a food that is not allowed, I then splurge and - eat other high calorie foods. | T | F |

Each question in this section is followed by a number of options. After reading each of the questions carefully, choose the option that best describes you and place an “X” over the answer.

| 1. How often are you dieting in a conscious effort to control your weight? | Rarely | Sometimes | Usually | Always |
| --- | --- | --- | --- | --- |
| 1. Would a weight fluctuation of 2 kilograms affect the way you live your life? | Not at all | Slightly | Moderately | Very much |
| 1. How often do you feel hungry? | Only at mealtimes | Sometimes between meals | Often between meals | Almost always |
| 1. Do your feelings of guilt about overeating help you control your food intake? | Never | Rarely | Often | Always |
| 1. How difficult would it be for you to stop eating halfway through dinner and not eat for the next four hours? | Easy | Slightly difficult | Moderately difficult | Very difficult |
| 1. How conscious are you of what you are eating? | Not at all | Slightly | Moderately | Extremely |
| 1. How frequently do you avoid “stocking up” on tempting foods? | Almost never | Seldom | Usually | Almost always |
| 1. How likely are you to shop for low calorie foods? | Unlikely | Slightly unlikely | Moderately likely | Very likely |
| 1. Do you eat sensibly in front of others and splurge alone? | Never | Rarely | Often | Always |
| 1. How likely are you to consciously eat slowly in order to cut down on how much you eat? | Unlikely | Slightly likely | Moderately likely | Very likely |
| 1. How frequently do you skip dessert because you are no longer hungry? | Rarely | Seldom | Al least once a week | Almost every day |
| 1. How likely are you to consciously eat less than you want? | Unlikely | Slightly likely | Moderately likely | Very likely |
| 1. Do you go on eating binges even when you’re not hungry? | Never | Rarely | Sometimes | At least once a week |
| 1. To what extent does this statement describe your eating behavior?   “I start dieting in the morning, but because of any number of things that happen during the day, by evening I have given up and eat what I want, promising myself to start dieting again tomorrow.” | Not like me | A little like me | Pretty good description of me | Describes me perfectly |
| 1. On a scale of 1 to 6, where 1 (one) means NO RESTRAINT in eating (eating whatever you want, whenever you want it) and 6 (six) means TOTAL RESTRAINT (constantly limiting food intake and never “giving in”), what number would you give yourself. Place an “X” over your answer.   1 - Eat whatever you want, whenever you want.  2 - Usually eat whatever you want, whenever you want.  3 - Often eat whatever you want, whenever you want.  4 - Often limit food intake, but often “give in”.  5 - Usually limit food intake, rarely “give in”.  6 - Constantly limiting food intake, never “giving in”. | | | | |

**7. Dyslipidemia**

| 7.1 **Have you ever been told by a doctor that you had high level of blood cholesterol?** | Yes …………………,,,………………….. 1  No ………………….…………………… 2  Doesn´t know …………………………… 9 | [____]  GO TO Q. 7.3 |
| --- | --- | --- |
| 7.2 **Have you received any treatment to lower high blood cholesterol level?**  **YOU CAN CHOOSE MORE THAN ONE ANSWER** | **Yes, Which one?**  Medicines……,,,,,,,……….......…………………1  Diet………………,,,,………….......…………….2  Increase in physical activity.................………….3  Home remedies………………………..…..…….4  No ………………………………………....……5 | [____]  [____]  [____] |
| 7.3 **Have you ever been told by a doctor that your triglyceride level was high?** | Yes ………………………...……………….. 1  No ……………………………….………… 2  Doesn´t know ……………………………… 9 | [____]  GO TO Q. 7.8 |
| 7.4 **Have you received any treatment to lower high triglycerides level?**  **YOU CAN CHOOSE MORE THAN ONE ANSWER** | **Yes**  Medicines………..………………………………1  Diet……………………...……………………….2  Increase in physical activity……….…………….3  No ………………………………………..………4 | [____]  [____]  [____] |

**8. List all current medications you are taking:**

Name Cause

_____________ ____________________________

_____________ ____________________________

_____________ ____________________________

_____________ ____________________________

_____________ ____________________________

_____________ ____________________________

_____________ ____________________________

**9.PHYSICAL ACTIVITY**

Think about all the **vigorous physical activities** that you did in the last 7 days. Vigorous physical activities refer to activities that take hard physical effort and make you breathe much harder than normal. Moderate activities refer to activities that take moderate physical effort and make you breathe somewhat harder than normal, this includes activities like aerobics, fast bicycling, heavy lifting, digging, farming, etc**. Think about only those physical activities that you did for at least 10 minutes at a time.**

| 9.1 ***On the last 7 days*, how many days did you do vigorous physical activities?** | No vigorous physical activities.………………..00  Refuses to answer……………………………....88  Doesn´t know……………………………..….....99  Mobility limitations or walking difficulties.…..55 | [____l____] Days per week GO TO Q. 9.4  4  GO TO Q. 9.12 |
| --- | --- | --- |
| 9.2 **How much time did you usually spend doing vigorous physical activities on one of those days?** | Refuses to answer…………………………..88  Doesn´t know………………………….…….99 | [____l____]  Hours per day  [____l____]  Minutes per day |
| 9.3 **How much time did you spend doing vigorous physical activities during the las 7 days?** | Refuses to answer ……………………..…..88  Doesn´t know…………………………..….99 | [____l____]  Hours per week  [____l____]  Minutes per week |

Think about all the **moderate activities** that you did in the last 7 days. Moderate activities refer to activities that make you breathe somewhat harder than normal, this includes activities like carrying light loads. cycling at a regular pace, etc. **Doesn´t include walking.**  **Think only about those physical activities that you did for at least 10 minutes at a time**.

| 9.4***During the last 7 days*, on how many days did you do moderate physical activities?** | No moderate physical activities ………....00  Refuses to answer …………………….….88  Doesn´t know………………………..….....99 | [____l____] Days per week GO TO Q. 9.7 |
| --- | --- | --- |
| 9.5 **How much time did you usually spend doing moderate physical activities on one of those days?** | Refuses to answer ………………………..…..88  Doesn´t know……………………….….…….99 | [____l____]  Hours per day  [____l____]  Minutes per day |
| 9.6 **How much time did you spend doing moderate physical activities during the last 7 days?** | Refuses to answer …………………………..88  Doesn´t know……………………………….99 | [____l____]  Hours per week  [____l____]  Minutes per week |

Now think of the **time that you spent walking during the last 7 days**. This includes walking at work, home, going from one place to another and/or any other walk you have taken solely for recreation, sport, exercise, or leisure. **Think only of activities in which you walked for at least 10 minutes at a time.**

| 9.7 ***During the last 7 days*, how many days did you walk at least 10 minutes in a row?** | No walking…………………00  Refuses to answer ………….88  Doesn´t know……………....99 | [____l____] Days per week GO TO Q. 9.10 |
| --- | --- | --- |
| 9.8. **How much time did you usually spend walking on one of those days?** | Refuses to answer…………..88  Doesn´t know……………….99 | [____l____]  Hours per day  [____l____]  Minutes per day |
| 9.9 **How much time did you spend walking during the last 7 days?** | Refuses to answer …………..88  Doesn´t know……………….99 | [____l____]  Hours per week  [____l____]  Minutes per week |

Now think on the **time you spend sitting during the last 7 days**. Include the time you spent sitting while at work, at home, while studying and during leisure time. **This may include the time you spend sitting** at a desk, visiting friends, reading, sitting or lying down to watch television.

| 9.10 ***During the last 7 days*, how much time did you spend sitting during one of those days?** | Refuses to answer…………..88  Doesn´t know……………….99 | [____l____]  Hours on a weekday  [____l____]  Minutes on a weekday |
| --- | --- | --- |
| 9.11 **How long were you sitting on Wednesday of last week?** | Refuses to answer …………..88  Doesn´t know……………….99 | [____l____]  Hours on Wednesday of last week  [____l____]  Minutes on Wednesday of last week |

9.12 From Monday to Friday, how many hours did you spend watching television. (including video games and movies)? _____

1. Never 4. 2-3 hours

2. Less than an hour 5. 4-6 hours

3. 1-2 hours 6. More than 6 hours

9.13 From Saturday to Sunday, how many hours did you spend watching television.(including video games and movies)?_____

1. Never 4. 2-3 hours

2. Less than an hour 5. 4-6 hours

3. 1-2 hours 6. More than 6 hours

9.14 From Monday to Friday, how many hours did you spend using the computer?_____

1. Never 4. 2-3 hours

2. Less than an hour 5. 4-6 hours

3. 1-2 hours 6. More than 6 hours

9.15 From Saturday to Sunday, how many hours did you spend using the computer?_____

1. Never 4. 2-3 hours

2. Less than an hour 5. 4-6 hours

3. 1-2 hours 6. More than 6 hours

**10. Diet behavior**

10.1 Describe your breakfast of the previous day

Food Quantity Preparation method

______________ ______________________ ___________________

______________ ______________________ ___________________

______________ ______________________ ___________________

______________ ______________________ ___________________

______________ ______________________ ___________________

______________ ______________________ ___________________

10.2 Describe your lunch of the previous day

Food Quantity Preparation method

______________ ______________________ ___________________

______________ ______________________ ___________________

______________ ______________________ ___________________

______________ ______________________ ___________________

______________ ______________________ ___________________

______________ ______________________ ___________________

10.3 Describe your dinner of the previous day

Food Quantity Preparation method

______________ ______________________ ___________________

______________ ______________________ ___________________

______________ ______________________ ___________________

______________ ______________________ ___________________

______________ ______________________ ___________________

______________ ______________________ ___________________

10.4 How often did you consume the following foods?

Fruit juice _____

1. Never or less than once a week 5. 5-6 times a week

2. 1-3 times a week 6. Once a day

3. Once a week 7. 2-3 times a day

4. 2-4 times a week 8. More than 3 times a day

10.5 Vegetables_____

1. Never or less than once a week 5. 5-6 times a week

2. 1-3 times a week 6. Once a day

3. Once a week 7. 2-3 times a day

4. 2-4 times a week 8. More than 3 times a day

10.6 Rice or pasta_____

1. Never or less than once a week 5. 5-6 times a week

2. 1-3 times a week 6. Once a day

3. Once a week 7. 2-3 times a day

4. 2-4 times a week 8. More than 3 times a day

10.7 Eggs_____

1. Never or less than once a week 5. 5-6 times a week

2. 1-3 times a week 6. Once a day

3. Once a week 7. 2-3 times a day

4. 2-4 times a week 8. More than 3 times a day

10.8 Tortillas_____

1. Never or less than once a week 5. 5-6 times a week

2. 1-3 times a week 6. Once a day

3. Once a week 7. 2-3 times a day

4. 2-4 times a week 8. More than 3 times a day

10.9 Baked goods_____

1. Never or less than once a week 5. 5-6 times a week

2. 1-3 times a week 6. Once a day

3. Once a week 7. 2-3 times a day

4. 2-4 times a week 8. More than 3 times a day

10.10 Beef_____

1. Never or less than once a week 5. 5-6 times a week

2. 1-3 times a week 6. Once a day

3. Once a week 7. 2-3 times a day

4. 2-4 times a week 8. More than 3 times a day

10.11 Chocolate_____

1. Never or less than once a week 5. 5-6 times a week

2. 1-3 times a week 6. Once a day

3. Once a week 7. 2-3 times a day

4. 2-4 times a week 8. More than 3 times a day

10.12 Soda (diet sodas are excluded)_____

1. Never or less than once a week 5. 5-6 times a week

2. 1-3 times a week 6. Once a day

3. Once a week 7. 2-3 times a day

4. 2-4 times a week 8. More than 3 times a day

10.13 Sweets_____

1. Never or less than once a week 5. 5-6 times a week

2. 1-3 times a week 6. Once a day

3. Once a week 7. 2-3 times a day

4. 2-4 times a week 8. More than 3 times a day

10.14 Take-out food _____

1. Never or less than once a week 5. 5-6 times a week

2. 1-3 times a week 6. Once a day

3. Once a week 7. 2-3 times a day

4. 2-4 times a week 8. More than 3 times a day

**11. Cardiovascular disease**

| 11.1 **Has a doctor told you that you have (or had) a/an…** | **Yes No**  **heart attack?** ...........................…............. 1 2  **angina pectoris/chest pain?** ..................... 1 2  **heart failure?** ............................................ 1 2  **another heart disease?** ............................ 1 2  Unstable angina………….………………. 1 2  Paralysis or a transient vision loss………. 1 2  Cerebral embolism...................................... 1 2  Aortic aneurysm……………………….… 1 2  Arterial insufficiency in the lower limbs... 1 2 | [____]  [____]  [____]  [____]  [____]  [____]  [____]  [____] |
| --- | --- | --- |

| **Interviewer: CONTINUE IF ANY OF THE ANSWERS IN THE PREVIOUS QUESTION IS “YES”, OTHERWISE GO TO QUESTION 12.** | | |
| --- | --- | --- |
| 11.2 **Were you hospitalized in order to treat the cardiovascular disease?** | Yes ................................................................ 1  No .............................................................. 2 | [____] |
| 11.3 **How old were you when you first suffered from a cardiovascular disease?** | Refuses to answer……………………………….88  Doesn´t know ………………………………….99 | [___\|___] Age in years |
| 11.4 **Are you currently taking any medication due to the cardiovascular disease?** | Yes ……………………………………...…. 1  No ………………………………………. 2  Refuses to answer………………………………… 8  Doesn´t know …........…………………………… 9 | [____] |

**12. Risk factors**

| 12.1 **Have you smoked at least 100 cigarettes (5 packs) in your entire life?** | Yes................................................... 1  No.................................................. 2  Has never smoked ........................... 3 | [____]    GO TO Q. 13 |
| --- | --- | --- |
| 12.2 **Do you currently smoke?** | Yes...................................................... 1  No.................................................... 2 | [____] |
| 12.3 **How many cigarettes and how often do you smoke (or smoked)?** | Daily.......................................................1  Weekly ...................................................2  Monthly ..................................................3  Occasional...............................................4  Refuses to answer. .................................8  Doesn´t know .........................................9 | [____]  **Frequency**  [___i___]  **Quantity** |
| 12.4 **How long have you been smoking (smoked) regularly?** | Less than a month ..................... 00  Refuses to answer............................... 88  Doesn´t know ……………................. 99 | **Months** [___i___]  **Years** [___i___] |
| 12.5 **Do you currently drink alcohol?** | Has never drank alcohol…………… ..........0  Yes ...............................................................1  No .................................................................2 | GO TO Q. 13  [____] |
| 12.6 On average, how many drinks and how often do you drink (or drank)? | Daily.......................................................1  Weekly ...................................................2  Monthly ..................................................3  Occasional...............................................4  Refuses to answer. .................................8  Doesn´t know .........................................9 | [____]  **Frequency**  [___i___]  **Quantity** |
| 12.7 How long have you been regularly drinking (or drank) that amount? | Less than a month .................................. 00  Refuses to answer …………………….. 88  Doesn´t know.........…………………… 99 | **Months** [___i___]  **Years** [___i___] |
| 12.8 Considering all types of alcoholic beverages, how many times during the past 30 days did you have 5 or more drinks on one time? | Currently sober………………. ...............98  Refuses to answer …………………….. 88  Doesn´t know.........……………………. 99 | [___i___]  **Times** |

**13. Thyroid Disorders**

| 13.1 Has any family member (grandparents, uncles, cousins, parents, siblings or children) ever had or has a thyroid gland disease? | Yes………………………………….……....1  No……………………………….……..…2  Doesn´t know……………………………………9 | [____] |
| --- | --- | --- |
| 13.2 Of the relatives who have had or have a thyroid disease, what was the manifestation? (you can indicate more than one) | Hiperthyroidism……………….………………..1  Hipothyroidism ……………………………...…2  Goitre (swelling of the thyroid gland)……...….3  Thyroid nodules ………………………………..4  Thyroid cancer …………………………..……..5  Doesn´t know/ refuses to answer….…………..99 | [____]  [____]  [____]  [____] |
| 13.3 Have you been diagnosed with a thyroid disease? | Yes………………………………….……....1  No……………………………….……..…2  Doesn´t know……………………………………9 | [____] |
| 13.4 If you have had any thyroid disease, what type is it? (you indicate more than one) | Hiperthyroidism……………….………………..1  Hipothyroidism ……………………………...…2  Goitre (swelling of the thyroid gland)……...….3  Thyroid nodules ………………………………..4  Thyroid cancer …………………………..……..5  Doesn´t know/ refuses to answer….…………..99 | [____]  [____]  [____]  [____] |
| 13.5 Have you ever received any thyroid treatment? | Yes………………………………….……....1  No……………………………….……..…2  Doesn´t know……………………………………9 | [____] |
| 13.6 Have you ever had a thyroid surgery? | Yes………………………………….……....1  No……………………………….……..…2  Doesn´t know……………………………………9 | [____] |
| 13.7 Have you ever received radioactive iodine? | Yes………………………………….……....1  No……………………………….……..…2  Doesn´t know……………………………………9 | [____] |
| 13.8 What is your current treatment for your thyroid condition? (you can indicate more than one): | Thyroid hormone therapy ……….……………1  Tapazole or Tiamazol………….………………2  Other……………………….…………………..3  (specify)____________________________  Doesn´t know…………………………………...9 | [____]  [____]  [____] |

**14. Mineral metabolism**

| 14.1 Have you ever broken or fractured any part of your body? | Yes………………………………….……....1  In which part of your body?_________________  No……………………………….……..…2  Doesn´t know……………………………………9 | [____] |
| --- | --- | --- |
| 14.2 Have any of your parents or siblings had a fracture? You can write more than one family member. | Yes………………………………….……....1  Who?______________________________  In which part of the body?___________________  Who?______________________________  In which part of the body?___________________  No……………………………….……..…2  Doesn´t know……………………………………9 | [____]  [____]  [____]  [____] |
| 14.3 Has a doctor told you that you had osteoporosis? | Yes………………………………….……....1  No……………………………….……..…2  Doesn´t know……………………………………9 | [____] |
| 14.4 Have any of your parents or siblings been diagnosed with osteoporosis? You can write more than one family member. | Yes………………………………….……....1  Who?______________________________  Who?______________________________  No……………………………….……..…2  Doesn´t know……………………………………9 | [____]  [____]  [____]  [____] |
| 14.5 Have you ever had kidney stones? | Yes………………………………….……....1  No……………………………….……..…2  Doesn´t know……………………………………9 | [____] |
| 14.6 Have you ever had high calcium levels in blood? | Yes………………………………….……....1  No……………………………….……..…2  Doesn´t know……………………………………9 | [____] |
| 14.7 Have you ever had chronic diarrhea or been diagnosed with malabsorption syndrome? | Yes………………………………….……....1  No……………………………….……..…2  Doesn´t know……………………………………9 | [____] |
| 14.8 From Monday through Friday of last week, how long were you exposed to sunlight? | Add the time of the 5 days | **Minutes** [___i___]  **Hours** [___i___] |

**15. Geriatric assessment**

| **Interviewer: CONTINUE IF THE PARTICIPANT IS AGED >50 YEARS, OTHERWISE GO TO QUESTION 16.** | | |
| --- | --- | --- |
| 15.1 Compared to others your age, how would you rate your health? | Very good ……………….. 1  Good …………………….. 2  Regular …………………... 3  Poor ……………………… 4  Refuses to answer/Doesn´t know ……99 | \|___\| |
| Activities of daily living |  |  |
| 15.2 Can you bathe totally by yourself (in shower, bath or with a bath sponge)? | Yes…………………………. 1  No ………………………... 2  Refuses to answer/Doesn´t know …... 99 | \|___\| |
| 15.3 Can you dress without help? Choose and change clothes by yourself (may need help in tying shoes) | Yes………………………. 1  No ……………………... 2  Refuses to answer/Doesn´t know …. 99 | \|___\| |
| 15.4 When you go to the bathroom, can you do it by yourself? Go to the toilet, get on and off, arrange clothes, and clean genital area without help (can use a walking stick or walker and use a bedpan or urinal at night) | Yes…………………………. 1  No ………………………... 2  Refuses to answer/Doesn´t know ….... 99 | \|___\| |
| 15.5 Can you move or travel by yourself? Moves in and out of bed or chair unassisted (can use a walking stick or walker) | Yes…………………………. 1  No ………………………... 2  Refuses to answer/Doesn´t know …… 99 | \|___\| |
| 15.6 Can you completely control your urination and bowel movements. (no sporadic accidents)? | Yes…………………………. 1  No ………………………... 2  Refuses to answer/Doesn´t know …... 99 | \|___\| |
| 15.7 Can you feed yourself and without help? Except for cutting meat or spreading butter on bread | Yes…………………………. 1  No ………………………... 2  Refuses to answer/Doesn´t know ….... 99 | \|___\| |
| Instrumental activities of daily living | | |
| 15.8 Can you prepare your own meals? | Without help ……………………...… 2  With some help ……………............... 1  Completely unable to prepare any meal…..…………………..…….…..... 0  Refuses to answer/Doesn´t know …... 99 | \|___\| |
| 15.9 Can you do your own housework? | Without help ………………………. 2  With some help …............................. 1  Completely unable to do any housework………………………….... 0  Refuses to answer/Doesn´t know …... 99 | \|___\| |
| 15.10 Can you do your own laundry? | Without help …………..…………… 2  With some help ………...……........... 1  Completely unable to do any laundry ……………………………………….. 0  Refuses to answer/Doesn´t know …... 99 | \|___\| |
| 15.11 Do you take your own medication? | Without help …………………..… 2  Needs some help ….............................. 1  Completely unable to take own medication ………………………….... 0  Refuses to answer/Doesn´t know …... 99 | \|___\| |
| 15.12 Can you get to places that are out of walking distance? | Without help …………………..…… 2  With some help ….............................. 1  Completely unable to travel ……..….. 0  Refuses to answer/Doesn´t know …... 99 | \|___\| |
| 15.13 Can you go shopping for groceries? | Without help ………………..……… 2  With some help ….............................. 1  Completely unable to do any shopping……………………………... 0  Refuses to answer/Doesn´t know …... 99 | \|___\| |
| 15.14 Can you manage your own money? | Without help ……………...………… 2  With some help ………………........... 1  Completely unable to handle money ... 0  Refuses to answer/Doesn´t know …... 99 | \|___\| |
| 15.15 Can you use the telephone? | Without help ……………..………… 2  With some help …………….............. 1  Completely unable to use the telephone………………..…………..... 0  Refuses to answer/Doesn´t know …... 99 | \|___\| |
| **Geriatric Depression Scale** | | |
| **Inteviewer: CHOOSE THE ANSWER THAT BEST DESCRIBES HOW THE PARTICIPANT FELT DURING THE LAST WEEK.**  **NOTE: THE SCORE OF EVERY QUESTION VARIES ACCORDING TO ITS NATURE.** | | |
| 15.16 Are you satisfied with your life? | Yes…………………………. 1  No ………………………... 0  Doesn´t know / refuses to answer ..…. 99 | \|___\| |
| 15.17 Have you stopped doing many of your activities and interests? | Yes…………………………. 1  No ………………………... 0  Doesn´t know / refuses to answer …... 99 | \|___\| |
| 15.18 Do you feel that your life is empty? | Yes…………………………. 1  No ………………………... 0  Doesn´t know / refuses to answer..…. 99 | \|___\| |
| 15.19 Do you often get bored? | Yes…………………………. 1  No ………………………... 0  Doesn´t know / refuses to answer …... 99 | \|___\| |
| 15.20 Are you in good spirits most of the time? | No …………………………. 1  Yes…………………………... 0  Doesn´t know / refuses to answer...…. 99 | \|___\| |
| 15.21 Are you afraid that something bad is going to happen to you? | Yes…………………………. 1  No ………………………... 0  Doesn´t know / refuses to answer …... 99 | \|___\| |
| 15.22 Do you feel happy most of the time? | No …………………………. 1  Yes…………………………... 0  Doesn´t know / refuses to answer….... 99 | \|___\| |
| 15.23 Do you often feel helpless? | Yes…………………………. 1  No ………………………... 0  Doesn´t know / refuses to answer..…. 99 | \|___\| |
| 15.24 Do you prefer to stay at home, rather than going out and doing new things? | Yes…………………………. 1  No ………………………... 0  Doesn´t know / refuses to answer ..…. 99 | \|___\| |
| 15.25 Do you feel you have more problems with memory than most? | Yes…………………………. 1  No ………………………... 0  Doesn´t know / refuses to answer ..…. 99 | \|___\| |
| 15.26 Do you think it is wonderful to be alive now? | No …………………………. 1  Yes…………………………... 0  Doesn´t know / refuses to answer .... 99 | \|___\| |
| 15.27 Do you feel worthless the way you are now? | Yes…………………………. 1  No ………………………... 0  Doesn´t know / refuses to answer …... 99 | \|___\| |
| 15.28 Do you feel full of energy? | No …………………………. 1  Yes…………………………... 0  Doesn´t know / refuses to answer…… 99 | \|___\| |
| 15.29 Do you feel that your situation is hopeless? | Yes……………….…………. 1  No ………………….……... 0  Doesn´t know / refuses to answer...…. 99 | \|___\| |
| 15.30 Do you think that most people are better off than you are? | Yes……………….…………. 1  No ………………….……... 0  Doesn´t know / refuses to answer...…. 99 | \|___\| |

PHYSICAL EXAMINATION

16. Was weight measurement performed?

Yes_____ _________kg_____gm

No_____ _________lbs

17. Was height measurement performed?

Yes_____ _________cm

No_____

18. Was waist circumference measurement performed?

Yes_____ _________cm

No____

19. Was hip circumference measurement performed?

Yes_____ _________cm

No_____

20. Was venous blood sample collected?

Yes_____ No_____

21. Was blood pressure measurement performed?

Yes_____ SBP_________ DBP _________

No___

**THREE YEAR ASSESSMENT**

**Was the participant located? Yes_____ No______ Why?___________________________**

**Date:______________________________________________________________________________**

**City**__________________________________________________________________________

Interviewer:____________________________________________________________

ADDRESS OR LOCATION DATA CHANGED: NO (CONTINUE) YES (UPDATE THE DATA BELOW)

_______________________________________________________________________________________________________________________________

(ADDRESS, INCLUDING STREET, HOUSE NUMBER, APARTMENT NUMBER, BUILDING, NEIGHBORHOOD, TOWNSHIP AND ZIP CODE)

PHONE NUMBER _____________________________________ E-MAIL___________________________________________________________

TWO CONTACT PEOPLE: (NAME, ADDRESS, INCLUDING STREET, HOUSE NUMBER, APARTMENT NUMBER, BUILDING, NEIGHBORHOOD, TOWNSHIP AND ZIP CODE, PHONE NUMBER AND E-MAIL)

1. ______________________________________________________________________________________________________________________
2. _______________________________________________________________________________________________________________________

**1. Socio-demographic background**

**1.1 Is the patient alive? Yes**: ____ DATE OF BIRTH: __/___/___ Current age:____

**No**_____

**1.2 Cause of death**:_________________________________________________________________________

**1.3 Document proving the cause of death**: _________________________________________

_______________________________________________________________________________________________

**1.4 Date of death**:_________________________________________________________________________

**1.5 Socioeconomic status______**

1. Low 2. Middle 3. High

**1.6. Highest degree or level of education completed**: ____

1. 1st grade 6. 6th grade 11. Never attended

2. 2nd grade 7. Middle school or technical program 12. Refuses to answer

3. 3rd grade 8. High school

4. 4th grade 9. Bachelor’s Degree

5. 5th grade 10. Master’s degree or higher

**1.6. Years of education:** ________

**1.8. Main occupation:** ___________________________________

**1.9 If female (under 50 years)**

**Number of pregnancies _________**

**Number of abortions _________ Number of deliveries _________**

**Age of first menstrual period ________ Age of last menstrual period ________**

**2. Family History:**

| 2.1 Have any of your parents, siblings, uncles, aunts or grandparents have or had diabetes? | Yes ………………........……….. 1  Who?____________________________  No.............................................. 2  Doesn´t know...................................... 9 | [____] |
| --- | --- | --- |
| 2.2 Have any of your parents, siblings, uncles, aunts or grandparents have or had obesity? | Yes ………………........……….. 1  Who?____________________________  No.............................................. 2  Doesn´t know...................................... 9 | [____] |
| 2.3 Have any of your parents, siblings, uncles, aunts or grandparents have or had high blood pressure? | Yes ………………........……….. 1  Who?____________________________  No.............................................. 2  Doesn´t know...................................... 9 | [____] |
| 2.4 Have any of your parents, siblings, uncles, aunts or grandparents have or had a heart attack? | Yes ………………........……….. 1  Who?____________________________  No.............................................. 2  Doesn´t know...................................... 9 | [____] |

**3. Diabetes**

| 3.1 **Have you ever been told by a doctor that you have diabetes or high blood sugar?** | Yes.....................................................................1  Yes, during pregnancy (gestational diabetes).....3  No………......................................................... 2 | | [____]  GO TO Q.4 |
| --- | --- | --- | --- |
| 3.2 **When were you first diagnosed with diabetes or with high blood sugar?** | Less than a month ……………... 00  Doesn´t know........................................ 99 | | **months** [___\|___]  **years**  [___\|___] |
| 3.3 **Have you been told to take prescribed medicine in order to control diabetes or lower blood sugar?** | Yes........................................... 1  No.......................................... 2 | | [____]  GO TO Q. 3.6 |
| 3.4 Are you currently taking pills or insulin to control blood sugar? | **Yes, Which one?**  Insulin........................................... 1  Pills................................................ 2  Both……………………………. 3  None........................................... 4 | | [____]  GO TO Q. 3.6  GO TO Q. 3.6 |
| 3.5 How many times and how frequently do you use insulin? | Daily……..…………………………… 1  Weekly…….………………….…….….2  Refuses to answer ...................................8  Doesn´t know ….........................................9 | | [___i___]  Times  [___]  **Frequency** |
| 3.6 **Are you currently undergoing other treatment in order to control your blood sugar?**  **YOU CAN CHOOSE MORE THAN ONE ANSWER** | **Yes, Which one?**  Diet.........................................................  Exercise.................................................  Homeopathic medicine......…….............  Herbal medicine.....................................  Hemotherapy...................……….….....  Other.............................……..............…  No……………………………………... | 01  02  03  04  05  77  07 | [___\|___]  [___\|___]  [___\|___]  [___\|___]  [___\|___]  [___\|___] |
| 3.7 **Because of diabetes, which of the following preventive measures had you followed to avoid complications?**  **YOU CAN CHOOSE MORE THAN ONE ANSWER** | Ophthalmologic examination ………………… 1  Take a daily aspirin pill.…………………...….. 2  Foot examination.…………………………...…..3  Renal function test...……………………….……4  None............................................................…….5  Other (specify)__________________________7 | | [____]  [____]  [____] |
| 3.8 **Because of diabetes have you…** | **Yes No**  **had leg or foot ulcers**  **that take more than 4 weeks to heal?**...….1 2  **had an amputation?**.................................... 1 2  **had decreased eyesight?**............................. 1 2  **had retinal damage.**.................................... 1 2  **lost your sight?**............................................ 1 2  **had a dialysis?**............................................. 1 2  **had a heart attack or stroke?**.......................................................... 1 2  **suffered from a diabetic coma?**................. 1 2  **lost feeling in any part of your body?**........1 2 | | [____]  [____]  [____]  [____]  [____]  [____]  [____]  [____]  [____] |

**4. Hypertension**

| 4.1 **Have you ever been told by a doctor that you had high blood pressure?** | Yes .......................................................... 1  No ........................................................ 2 | | [____] |
| --- | --- | --- | --- |
| **Interviewer: CONTINUE ONLY IF THE PARTICIPANT IS A WOMAN, OTHERWISE GO TO Q. 4.5** | | | |
| 4.2 **Were you pregnant when you were told to had high blood pressure?** | Yes .................................................................... 1  No ..................................................................... 2 | | [____] |
| 4.3 **Has a doctor or health professional ever diagnosed you with hypertension only during pregnancy? (preeclampsia)** | Yes .................................................................... 1  No ...................................................................... 2 | | [____] |
| 4.4 **Have you had a baby that weighed over 3.5 kilograms or 7.7 lbs. at birth?** | Yes ………………........………………………. 1  No....................................................................... 2  Doesn´t know......................................................... 9 | | [____] |
| 4.5 **How long ago did a doctor told you to had high blood pressure?** | I haven’t been diagnosed with hypertension........ 00  **(GO TO Q.5)**  Less than a month ................................................. 01  Doesn´t know............................................................ 99 | | **Months** [___\|___]  **Years** [___\|___] |
| 4.6 **Are you currently taking pills in order to control your blood pressure?** | Yes ....................................................................... 1  No ......................................................................... 2  Doesn´t know ........................................................... 9 | | [____]  GO TO Q. 4.8 |
| 4.7 H**ow long have you been taking these pills?** | Less than a month ......................................................00  Doesn´t know…………………………………….……99 | | **Months** [___\|___]  **Years** [___\|___] |
| 4.8 **Where do you go for your blood pressure check-ups?** | IMSS OPORTUNIDADES ....…………………......01  IMSS ........................................................................02  Secretaría de Salud....................................................03  SEGURO POPULAR (SSA)....................................04  DIF, CRUZ ROJA, INI ……....................................05  ISSSTE, ISSSTE ESTATAL ..................................06  MARINA/DEFENSA, PEMEX ..............................07  PARTICULAR ........................................................08  HOSPITAL CIVIL ..................................................09  NATIONAL INSTITUTES …................................10  OTHER ………………...........................................77  DOESN´T KNOW........................................................99 | | [___\|___] |
| 4.9 **Are you currently undergoing other treatment to control your high blood pressure?** | **If yes, which of the following?**  Diet ...........................................................................1  Exercise .................................................................2  Homeopathy.................................................……..3  Herbal remedies ............................................……4  Reduce sodium intake……....................................5  Other ..……………………………………...……7  No ........................…...........………….....……….6 | | [____]  [____]  [____]  [____]  [____]  [____] |
| 4.10 **How many times and how frequently did you or someone else checked your blood pressure?** | Weekly ................................................  Monthly ..............................................  Annually...................................................  Doesn´t know.........................................  Doesn’t do it……................................... | 1  2  3  8  4 | [____]  **Frequency**  [___\|___]  **Times** |

**5. Obesity**

| 5.1 **Has a doctor/ dietitian/ nutritionist ever told you that you are or were overweight?** | Yes………………………………..…………..1  No…………………………………………..…2  Doesn´t know………..…………………......…9 | [____]  GO TO Q. 6 |
| --- | --- | --- |
| 5.2 **How old were you when you were told you had obesity?** | Refuses to answer.............................................. 88  Doesn´t know ……………................................ 99 | [___i___]  **Age in years** |
| 5.3 **What has been your maximum weight** (without pregnancy)? | Refuses to answer.............................................. 88  Doesn´t know ……………................................ 99 | [___\|___]  **kilograms** |
| 5.4 **Did you followed any treatment to control your weight?** | Yes………………………………….…………....1  No……………………………………………..…2  Doesn´t know………………………………...…..9 | [____] |
| 5.5 **During last year, have you lost or gained weight?** | **Yes**  Gained weight ……………………………………1  Lost weight ………………………………….........2  Answers 3 to 6  GO TO Q. 6  No weight change ………………………………...3  Gained or lost weight due to pregnancy……...…...4  Refuses to answer…..…………………..…………8  Doesn´t know…………………………-…..………9 | [____] |
| 5.6 **How many kilograms or pounds did you gain (or lost)?** | GO TO Q. 6  Gained weight | [___\|___]  **Kilograms or lbs.** |
| **CONTINUE IF YOU LOST MORE THAN 5 KILOGRAMS, IF YOU LOST LESS THAN 5 KILOGRAMS GO TO QUESTION 6** | | |
| 5.6 **Was this weight loss intentional?** | Yes……………………………………………..1  No ……………………………………………...2  Refuses to answer ………………….………….8  Doesn´t know…………….……….……………9 | [____]  GO TO Q. 6 |
| 5.7 **What did you do to lose weight?**  **YOU CAN CHOOSE MORE THAN ONE ANSWER** | Ate less food………………..……..………………….01  Ate low calorie foods……………………………..….02  Ate low fat foods …………………………………….03  Exercised…………………….....………………….....04  Skipped meals….……………………..……………...05  Ate “diet” products …………………………...……...06  Used a weight loss liquid diet formula (i.e. SlimFast) 07  Participated in a weight loss program………..……….08  Followed a special diet announced on a magazine, internet, television or recommended by friends or family............................................................................09  Followed a special diet prescribed by a doctor/ dietitian/ nutritionist…………………………….........................10  Took diet pills prescribed by a doctor ………………..11  Took other medicines, herbs, supplements, or homeopathic medicine not needing a prescription……12  Increased physical activity…………….……………...13  Drank lots of water………...…………………………14  Other (specify) _______________________________77  Doesn´t know………………………………………....88  Refuses to answer…….……………………………….99 | [___\|___]  [___\|___]  [___\|___]  [___\|___]  [___\|___]  [___\|___] |

|  | True | False |
| --- | --- | --- |
| 1. When I smell a sizzling steak or see a juicy piece of meat, I find it very difficult to keep from eating, even if I have just finished a meal | T | F |
| 1. I usually eat too much at social situations, like parties and picnics. | T | F |
| 1. I am usually so hungry that I eat more than three times a day. | T | F |
| 1. When I have eaten my calorie limit, I am usually good about not eating any more. | T | F |
| 1. Dieting is so hard for me because I get too hungry. | T | F |
| 1. I deliberately take small helpings as a means of controlling my weight. | T | F |
| 1. Sometimes things just taste so good that I keep on eating, even when I am no longer hungry. | T | F |
| 1. Since I am often hungry, I sometimes wish that while I am eating, an expert would tell me that I have had enough or that I can have something more to eat. | T | F |
| 1. When I feel anxious, I find myself eating. | T | F |
| 1. Life is too short to worry about dieting. | T | F |
| 1. Since my weight goes up and down, I have gone on reducing diets more than once. | T | F |
| 1. I often feel so hungry that I just have to eat something. | T | F |
| 1. When I am with someone who is overeating, I usually overeat too. | T | F |
| 1. I have a pretty good idea of the number of calories in common food. | T | F |
| 1. Sometimes when I start eating, I just can’t stop. | T | F |
| 1. It is not difficult for me to leave something on my plate. | T | F |
| 1. At certain times of the day, I get hungry because I have gotten used to eating then. | T | F |
| 1. While on a diet, if I eat food that is not allowed, I consciously eat less for a period of time to make up for it. | T | F |
| 1. Being with someone who is eating often makes me hungry enough to also eat. | T | F |
| 1. When I feel blue, I often overeat. | T | F |
| 1. I enjoy eating too much to spoil it by counting calories or watching my weight. | T | F |
| 1. When I see a real delicacy, I often get so hungry that I have to eat right away. | T | F |
| 1. I often stop eating when I am not really full as a conscious means of limiting the amount that I eat. | T | F |
| 1. I get so hungry that my stomach often seems like a bottomless pit. | T | F |
| 1. My weight has hardly changed at all in the last ten years. | T | F |
| 1. I am always hungry, so it is hard for me to stop eating before I finish the food on my plate. | T | F |
| 1. When I feel lonely, I console myself by eating. | T | F |
| 1. I consciously hold back at meals in order not to gain weight. | T | F |
| 1. I sometimes get very hungry late in the evening or at night. | T | F |
| 1. I eat anything I want, any time I want. | T | F |
| 1. Without even thinking about it, I take a long time to eat. | T | F |
| 1. I count calories as a conscious means of controlling my weight. | T | F |
| 1. I do not eat some foods because they make me fat. | T | F |
| 1. I am always hungry enough to eat at any time. | T | F |
| 1. I pay a great deal of attention to changes in my figure. | T | F |
| 1. While on a diet, if I eat a food that is not allowed, I then splurge and - eat other high calorie foods. | T | F |

Each question in this section is followed by a number of options. After reading each of the questions carefully, choose the option that best describes you and place an “X” over the answer.

| 1. How often are you dieting in a conscious effort to control your weight? | Rarely | Sometimes | Usually | Always |
| --- | --- | --- | --- | --- |
| 1. Would a weight fluctuation of 2 kilograms affect the way you live your life? | Not at all | Slightly | Moderately | Very much |
| 1. How often do you feel hungry? | Only at mealtimes | Sometimes between meals | Often between meals | Almost always |
| 1. Do your feelings of guilt about overeating help you control your food intake? | Never | Rarely | Often | Always |
| 1. How difficult would it be for you to stop eating halfway through dinner and not eat for the next four hours? | Easy | Slightly difficult | Moderately difficult | Very difficult |
| 1. How conscious are you of what you are eating? | Not at all | Slightly | Moderately | Extremely |
| 1. How frequently do you avoid “stocking up” on tempting foods? | Almost never | Seldom | Usually | Almost always |
| 1. How likely are you to shop for low calorie foods? | Unlikely | Slightly unlikely | Moderately likely | Very likely |
| 1. Do you eat sensibly in front of others and splurge alone? | Never | Rarely | Often | Always |
| 1. How likely are you to consciously eat slowly in order to cut down on how much you eat? | Unlikely | Slightly likely | Moderately likely | Very likely |
| 1. How frequently do you skip dessert because you are no longer hungry? | Rarely | Seldom | Al least once a week | Almost every day |
| 1. How likely are you to consciously eat less than you want? | Unlikely | Slightly likely | Moderately likely | Very likely |
| 1. Do you go on eating binges even when you’re not hungry? | Never | Rarely | Sometimes | At least once a week |
| 1. To what extent does this statement describe your eating behavior?   “I start dieting in the morning, but because of any number of things that happen during the day, by evening I have given up and eat what I want, promising myself to start dieting again tomorrow.” | Not like me | A little like me | Pretty good description of me | Describes me perfectly |
| 1. On a scale of 1 to 6, where 1 (one) means NO RESTRAINT in eating (eating whatever you want, whenever you want it) and 6 (six) means TOTAL RESTRAINT (constantly limiting food intake and never “giving in”), what number would you give yourself. Place an “X” over your answer.   1 - Eat whatever you want, whenever you want.  2 - Usually eat whatever you want, whenever you want.  3 - Often eat whatever you want, whenever you want.  4 - Often limit food intake, but often “give in”.  5 - Usually limit food intake, rarely “give in”.  6 - Constantly limiting food intake, never “giving in”. | | | | |

**7. Dyslipidemia**

| 7.1 **Have you ever been told by a doctor that you had high level of blood cholesterol?** | Yes …………………,,,………………….. 1  No ………………….…………………… 2  Doesn´t know …………………………… 9 | [____]  GO TO Q. 7.3 |
| --- | --- | --- |
| 7.2 **Have you received any treatment to lower high blood cholesterol level?**  **YOU CAN CHOOSE MORE THAN ONE ANSWER** | **Yes, Which one?**  Medicines……,,,,,,,……….......…………………1  Diet………………,,,,………….......…………….2  Increase in physical activity.................………….3  Home remedies………………………..…..…….4  No ………………………………………....……5 | [____]  [____]  [____] |
| 7.3 **Have you ever been told by a doctor that your triglyceride level was high?** | Yes ………………………...……………….. 1  No ……………………………….………… 2  Doesn´t know ……………………………… 9 | [____]  GO TO Q. 7.8 |
| 7.4 **Have you received any treatment to lower high triglycerides level?**  **YOU CAN CHOOSE MORE THAN ONE ANSWER** | **Yes**  Medicines………..………………………………1  Diet……………………...……………………….2  Increase in physical activity……….…………….3  No ………………………………………..………4 | [____]  [____]  [____] |

**8. List all current medications you are taking:**

Name Cause

_____________ ____________________________

_____________ ____________________________

_____________ ____________________________

_____________ ____________________________

_____________ ____________________________

_____________ ____________________________

_____________ ____________________________

**9.PHYSICAL ACTIVITY**

Think about all the **vigorous physical activities** that you did in the last 7 days. Vigorous physical activities refer to activities that take hard physical effort and make you breathe much harder than normal. Moderate activities refer to activities that take moderate physical effort and make you breathe somewhat harder than normal, this includes activities like aerobics, fast bicycling, heavy lifting, digging, farming, etc**. Think about only those physical activities that you did for at least 10 minutes at a time.**

| 9.1 ***On the last 7 days*, how many days did you do vigorous physical activities?** | No vigorous physical activities.………………..00  Refuses to answer……………………………....88  Doesn´t know……………………………..….....99  Mobility limitations or walking difficulties.…..55 | [____l____] Days per week GO TO Q. 9.4  4  GO TO Q. 9.12 |
| --- | --- | --- |
| 9.2 **How much time did you usually spend doing vigorous physical activities on one of those days?** | Refuses to answer…………………………..88  Doesn´t know………………………….…….99 | [____l____]  Hours per day  [____l____]  Minutes per day |
| 9.3 **How much time did you spend doing vigorous physical activities during the las 7 days?** | Refuses to answer ……………………..…..88  Doesn´t know…………………………..….99 | [____l____]  Hours per week  [____l____]  Minutes per week |

Think about all the **moderate activities** that you did in the last 7 days. Moderate activities refer to activities that make you breathe somewhat harder than normal, this includes activities like carrying light loads. cycling at a regular pace, etc. **Doesn´t include walking.**  **Think only about those physical activities that you did for at least 10 minutes at a time**.

| 9.4***During the last 7 days*, on how many days did you do moderate physical activities?** | No moderate physical activities ………....00  Refuses to answer …………………….….88  Doesn´t know………………………..….....99 | [____l____] Days per week GO TO Q. 9.7 |
| --- | --- | --- |
| 9.5 **How much time did you usually spend doing moderate physical activities on one of those days?** | Refuses to answer ………………………..…..88  Doesn´t know……………………….….…….99 | [____l____]  Hours per day  [____l____]  Minutes per day |
| 9.6 **How much time did you spend doing moderate physical activities during the last 7 days?** | Refuses to answer …………………………..88  Doesn´t know……………………………….99 | [____l____]  Hours per week  [____l____]  Minutes per week |

Now think of the **time that you spent walking during the last 7 days**. This includes walking at work, home, going from one place to another and/or any other walk you have taken solely for recreation, sport, exercise, or leisure. **Think only of activities in which you walked for at least 10 minutes at a time.**

| 9.7 ***During the last 7 days*, how many days did you walk at least 10 minutes in a row?** | No walking…………………00  Refuses to answer ………….88  Doesn´t know……………....99 | [____l____] Days per week GO TO Q. 9.10 |
| --- | --- | --- |
| 9.8. **How much time did you usually spend walking on one of those days?** | Refuses to answer…………..88  Doesn´t know……………….99 | [____l____]  Hours per day  [____l____]  Minutes per day |
| 9.9 **How much time did you spend walking during the last 7 days?** | Refuses to answer …………..88  Doesn´t know……………….99 | [____l____]  Hours per week  [____l____]  Minutes per week |

Now think on the **time you spend sitting during the last 7 days**. Include the time you spent sitting while at work, at home, while studying and during leisure time. **This may include the time you spend sitting** at a desk, visiting friends, reading, sitting or lying down to watch television.

| 9.10 ***During the last 7 days*, how much time did you spend sitting during one of those days?** | Refuses to answer…………..88  Doesn´t know……………….99 | [____l____]  Hours on a weekday  [____l____]  Minutes on a weekday |
| --- | --- | --- |
| 9.11 **How long were you sitting on Wednesday of last week?** | Refuses to answer …………..88  Doesn´t know……………….99 | [____l____]  Hours on Wednesday of last week  [____l____]  Minutes on Wednesday of last week |

9.12 From Monday to Friday, how many hours did you spend watching television. (including video games and movies)? _____

1. Never 4. 2-3 hours

2. Less than an hour 5. 4-6 hours

3. 1-2 hours 6. More than 6 hours

9.13 From Saturday to Sunday, how many hours did you spend watching television.(including video games and movies)?_____

1. Never 4. 2-3 hours

2. Less than an hour 5. 4-6 hours

3. 1-2 hours 6. More than 6 hours

9.14 From Monday to Friday, how many hours did you spend using the computer?_____

1. Never 4. 2-3 hours

2. Less than an hour 5. 4-6 hours

3. 1-2 hours 6. More than 6 hours

9.15 From Saturday to Sunday, how many hours did you spend using the computer?_____

1. Never 4. 2-3 hours

2. Less than an hour 5. 4-6 hours

3. 1-2 hours 6. More than 6 hours

**10. Diet behavior**

10.1 Describe your breakfast of the previous day

Food Quantity Preparation method

______________ ______________________ ___________________

______________ ______________________ ___________________

______________ ______________________ ___________________

______________ ______________________ ___________________

______________ ______________________ ___________________

______________ ______________________ ___________________

10.2 Describe your lunch of the previous day

Food Quantity Preparation method

______________ ______________________ ___________________

______________ ______________________ ___________________

______________ ______________________ ___________________

______________ ______________________ ___________________

______________ ______________________ ___________________

______________ ______________________ ___________________

10.3 Describe your dinner of the previous day

Food Quantity Preparation method

______________ ______________________ ___________________

______________ ______________________ ___________________

______________ ______________________ ___________________

______________ ______________________ ___________________

______________ ______________________ ___________________

______________ ______________________ ___________________

10.4 How often did you consume the following foods?

Fruit juice _____

1. Never or less than once a week 5. 5-6 times a week

2. 1-3 times a week 6. Once a day

3. Once a week 7. 2-3 times a day

4. 2-4 times a week 8. More than 3 times a day

10.5 Vegetables_____

1. Never or less than once a week 5. 5-6 times a week

2. 1-3 times a week 6. Once a day

3. Once a week 7. 2-3 times a day

4. 2-4 times a week 8. More than 3 times a day

10.6 Rice or pasta_____

1. Never or less than once a week 5. 5-6 times a week

2. 1-3 times a week 6. Once a day

3. Once a week 7. 2-3 times a day

4. 2-4 times a week 8. More than 3 times a day

10.7 Eggs_____

1. Never or less than once a week 5. 5-6 times a week

2. 1-3 times a week 6. Once a day

3. Once a week 7. 2-3 times a day

4. 2-4 times a week 8. More than 3 times a day

10.8 Tortillas_____

1. Never or less than once a week 5. 5-6 times a week

2. 1-3 times a week 6. Once a day

3. Once a week 7. 2-3 times a day

4. 2-4 times a week 8. More than 3 times a day

10.9 Baked goods_____

1. Never or less than once a week 5. 5-6 times a week

2. 1-3 times a week 6. Once a day

3. Once a week 7. 2-3 times a day

4. 2-4 times a week 8. More than 3 times a day

10.10 Beef_____

1. Never or less than once a week 5. 5-6 times a week

2. 1-3 times a week 6. Once a day

3. Once a week 7. 2-3 times a day

4. 2-4 times a week 8. More than 3 times a day

10.11 Chocolate_____

1. Never or less than once a week 5. 5-6 times a week

2. 1-3 times a week 6. Once a day

3. Once a week 7. 2-3 times a day

4. 2-4 times a week 8. More than 3 times a day

10.12 Soda (diet sodas are excluded)_____

1. Never or less than once a week 5. 5-6 times a week

2. 1-3 times a week 6. Once a day

3. Once a week 7. 2-3 times a day

4. 2-4 times a week 8. More than 3 times a day

10.13 Sweets_____

1. Never or less than once a week 5. 5-6 times a week

2. 1-3 times a week 6. Once a day

3. Once a week 7. 2-3 times a day

4. 2-4 times a week 8. More than 3 times a day

10.14 Take-out food _____

1. Never or less than once a week 5. 5-6 times a week

2. 1-3 times a week 6. Once a day

3. Once a week 7. 2-3 times a day

4. 2-4 times a week 8. More than 3 times a day

**11. Cardiovascular disease**

| 11.1 **Has a doctor told you that you have (or had) a/an…** | **Yes No**  **heart attack?** ...........................…............. 1 2  **angina pectoris/chest pain?** ..................... 1 2  **heart failure?** ............................................ 1 2  **another heart disease?** ............................ 1 2  Unstable angina………….………………. 1 2  Paralysis or a transient vision loss………. 1 2  Cerebral embolism...................................... 1 2  Aortic aneurysm……………………….… 1 2  Arterial insufficiency in the lower limbs... 1 2 | [____]  [____]  [____]  [____]  [____]  [____]  [____]  [____] |
| --- | --- | --- |

| **Interviewer: CONTINUE IF ANY OF THE ANSWERS IN THE PREVIOUS QUESTION IS “YES”, OTHERWISE GO TO QUESTION 12.** | | |
| --- | --- | --- |
| 11.2 **Were you hospitalized in order to treat the cardiovascular disease?** | Yes ................................................................ 1  No .............................................................. 2 | [____] |
| 11.3 **How old were you when you first suffered from a cardiovascular disease?** | Refuses to answer……………………………….88  Doesn´t know ………………………………….99 | [___\|___] Age in years |
| 11.4 **Are you currently taking any medication due to the cardiovascular disease?** | Yes ……………………………………...…. 1  No ………………………………………. 2  Refuses to answer………………………………… 8  Doesn´t know …........…………………………… 9 | [____] |

**12. Risk factors**

| 12.1 **Have you smoked at least 100 cigarettes (5 packs) in your entire life?** | Yes................................................... 1  No.................................................. 2  Has never smoked ........................... 3 | [____]    GO TO Q. 13 |
| --- | --- | --- |
| 12.2 **Do you currently smoke?** | Yes...................................................... 1  No.................................................... 2 | [____] |
| 12.3 **How many cigarettes and how often do you smoke (or smoked)?** | Daily.......................................................1  Weekly ...................................................2  Monthly ..................................................3  Occasional...............................................4  Refuses to answer. .................................8  Doesn´t know .........................................9 | [____]  **Frequency**  [___i___]  **Quantity** |
| 12.4 **How long have you been smoking (smoked) regularly?** | Less than a month ..................... 00  Refuses to answer............................... 88  Doesn´t know ……………................. 99 | **Months** [___i___]  **Years** [___i___] |
| 12.5 **Do you currently drink alcohol?** | Has never drank alcohol…………… ..........0  Yes ...............................................................1  No .................................................................2 | GO TO Q. 13  [____] |
| 12.6 On average, how many drinks and how often do you drink (or drank)? | Daily.......................................................1  Weekly ...................................................2  Monthly ..................................................3  Occasional...............................................4  Refuses to answer. .................................8  Doesn´t know .........................................9 | [____]  **Frequency**  [___i___]  **Quantity** |
| 12.7 How long have you been regularly drinking (or drank) that amount? | Less than a month .................................. 00  Refuses to answer …………………….. 88  Doesn´t know.........…………………… 99 | **Months** [___i___]  **Years** [___i___] |
| 12.8 Considering all types of alcoholic beverages, how many times during the past 30 days did you have 5 or more drinks on one time? | Currently sober………………. ...............98  Refuses to answer …………………….. 88  Doesn´t know.........……………………. 99 | [___i___]  **Times** |

**13. Thyroid Disorders**

| 13.1 Has any family member (grandparents, uncles, cousins, parents, siblings or children) ever had or has a thyroid gland disease? | Yes………………………………….……....1  No……………………………….……..…2  Doesn´t know……………………………………9 | [____] |
| --- | --- | --- |
| 13.2 Of the relatives who have had or have a thyroid disease, what was the manifestation? (you can indicate more than one) | Hiperthyroidism……………….………………..1  Hipothyroidism ……………………………...…2  Goitre (swelling of the thyroid gland)……...….3  Thyroid nodules ………………………………..4  Thyroid cancer …………………………..……..5  Doesn´t know/ refuses to answer….…………..99 | [____]  [____]  [____]  [____] |
| 13.3 Have you been diagnosed with a thyroid disease? | Yes………………………………….……....1  No……………………………….……..…2  Doesn´t know……………………………………9 | [____] |
| 13.4 If you have had any thyroid disease, what type is it? (you indicate more than one) | Hiperthyroidism……………….………………..1  Hipothyroidism ……………………………...…2  Goitre (swelling of the thyroid gland)……...….3  Thyroid nodules ………………………………..4  Thyroid cancer …………………………..……..5  Doesn´t know/ refuses to answer….…………..99 | [____]  [____]  [____]  [____] |
| 13.5 Have you ever received any thyroid treatment? | Yes………………………………….……....1  No……………………………….……..…2  Doesn´t know……………………………………9 | [____] |
| 13.6 Have you ever had a thyroid surgery? | Yes………………………………….……....1  No……………………………….……..…2  Doesn´t know……………………………………9 | [____] |
| 13.7 Have you ever received radioactive iodine? | Yes………………………………….……....1  No……………………………….……..…2  Doesn´t know……………………………………9 | [____] |
| 13.8 What is your current treatment for your thyroid condition? (you can indicate more than one): | Thyroid hormone therapy ……….……………1  Tapazole or Tiamazol………….………………2  Other……………………….…………………..3  (specify)____________________________  Doesn´t know…………………………………...9 | [____]  [____]  [____] |

**14. Mineral metabolism**

| 14.1 Have you ever broken or fractured any part of your body? | Yes………………………………….……....1  In which part of your body?_________________  No……………………………….……..…2  Doesn´t know……………………………………9 | [____] |
| --- | --- | --- |
| 14.2 Have any of your parents or siblings had a fracture? You can write more than one family member. | Yes………………………………….……....1  Who?______________________________  In which part of the body?___________________  Who?______________________________  In which part of the body?___________________  No……………………………….……..…2  Doesn´t know……………………………………9 | [____]  [____]  [____]  [____] |
| 14.3 Has a doctor told you that you had osteoporosis? | Yes………………………………….……....1  No……………………………….……..…2  Doesn´t know……………………………………9 | [____] |
| 14.4 Have any of your parents or siblings been diagnosed with osteoporosis? You can write more than one family member. | Yes………………………………….……....1  Who?______________________________  Who?______________________________  No……………………………….……..…2  Doesn´t know……………………………………9 | [____]  [____]  [____]  [____] |
| 14.5 Have you ever had kidney stones? | Yes………………………………….……....1  No……………………………….……..…2  Doesn´t know……………………………………9 | [____] |
| 14.6 Have you ever had high calcium levels in blood? | Yes………………………………….……....1  No……………………………….……..…2  Doesn´t know……………………………………9 | [____] |
| 14.7 Have you ever had chronic diarrhea or been diagnosed with malabsorption syndrome? | Yes………………………………….……....1  No……………………………….……..…2  Doesn´t know……………………………………9 | [____] |
| 14.8 From Monday through Friday of last week, how long were you exposed to sunlight? | Add the time of the 5 days | **Minutes** [___i___]  **Hours** [___i___] |

**15. Geriatric assessment**

| **Interviewer: CONTINUE IF THE PARTICIPANT IS AGED >50 YEARS, OTHERWISE GO TO QUESTION 16.** | | |
| --- | --- | --- |
| 15.1 Compared to others your age, how would you rate your health? | Very good ……………….. 1  Good …………………….. 2  Regular …………………... 3  Poor ……………………… 4  Refuses to answer/Doesn´t know ……99 | \|___\| |
| Activities of daily living |  |  |
| 15.2 Can you bathe totally by yourself (in shower, bath or with a bath sponge)? | Yes…………………………. 1  No ………………………... 2  Refuses to answer/Doesn´t know …... 99 | \|___\| |
| 15.3 Can you dress without help? Choose and change clothes by yourself (may need help in tying shoes) | Yes………………………. 1  No ……………………... 2  Refuses to answer/Doesn´t know …. 99 | \|___\| |
| 15.4 When you go to the bathroom, can you do it by yourself? Go to the toilet, get on and off, arrange clothes, and clean genital area without help (can use a walking stick or walker and use a bedpan or urinal at night) | Yes…………………………. 1  No ………………………... 2  Refuses to answer/Doesn´t know ….... 99 | \|___\| |
| 15.5 Can you move or travel by yourself? Moves in and out of bed or chair unassisted (can use a walking stick or walker) | Yes…………………………. 1  No ………………………... 2  Refuses to answer/Doesn´t know …… 99 | \|___\| |
| 15.6 Can you completely control your urination and bowel movements. (no sporadic accidents)? | Yes…………………………. 1  No ………………………... 2  Refuses to answer/Doesn´t know …... 99 | \|___\| |
| 15.7 Can you feed yourself and without help? Except for cutting meat or spreading butter on bread | Yes…………………………. 1  No ………………………... 2  Refuses to answer/Doesn´t know ….... 99 | \|___\| |
| Instrumental activities of daily living | | |
| 15.8 Can you prepare your own meals? | Without help ……………………...… 2  With some help ……………............... 1  Completely unable to prepare any meal…..…………………..…….…..... 0  Refuses to answer/Doesn´t know …... 99 | \|___\| |
| 15.9 Can you do your own housework? | Without help ………………………. 2  With some help …............................. 1  Completely unable to do any housework………………………….... 0  Refuses to answer/Doesn´t know …... 99 | \|___\| |
| 15.10 Can you do your own laundry? | Without help …………..…………… 2  With some help ………...……........... 1  Completely unable to do any laundry ……………………………………….. 0  Refuses to answer/Doesn´t know …... 99 | \|___\| |
| 15.11 Do you take your own medication? | Without help …………………..… 2  Needs some help ….............................. 1  Completely unable to take own medication ………………………….... 0  Refuses to answer/Doesn´t know …... 99 | \|___\| |
| 15.12 Can you get to places that are out of walking distance? | Without help …………………..…… 2  With some help ….............................. 1  Completely unable to travel ……..….. 0  Refuses to answer/Doesn´t know …... 99 | \|___\| |
| 15.13 Can you go shopping for groceries? | Without help ………………..……… 2  With some help ….............................. 1  Completely unable to do any shopping……………………………... 0  Refuses to answer/Doesn´t know …... 99 | \|___\| |
| 15.14 Can you manage your own money? | Without help ……………...………… 2  With some help ………………........... 1  Completely unable to handle money ... 0  Refuses to answer/Doesn´t know …... 99 | \|___\| |
| 15.15 Can you use the telephone? | Without help ……………..………… 2  With some help …………….............. 1  Completely unable to use the telephone………………..…………..... 0  Refuses to answer/Doesn´t know …... 99 | \|___\| |
| **Geriatric Depression Scale** | | |
| **Inteviewer: CHOOSE THE ANSWER THAT BEST DESCRIBES HOW THE PARTICIPANT FELT DURING THE LAST WEEK.**  **NOTE: THE SCORE OF EVERY QUESTION VARIES ACCORDING TO ITS NATURE.** | | |
| 15.16 Are you satisfied with your life? | Yes…………………………. 1  No ………………………... 0  Doesn´t know / refuses to answer ..…. 99 | \|___\| |
| 15.17 Have you stopped doing many of your activities and interests? | Yes…………………………. 1  No ………………………... 0  Doesn´t know / refuses to answer …... 99 | \|___\| |
| 15.18 Do you feel that your life is empty? | Yes…………………………. 1  No ………………………... 0  Doesn´t know / refuses to answer..…. 99 | \|___\| |
| 15.19 Do you often get bored? | Yes…………………………. 1  No ………………………... 0  Doesn´t know / refuses to answer …... 99 | \|___\| |
| 15.20 Are you in good spirits most of the time? | No …………………………. 1  Yes…………………………... 0  Doesn´t know / refuses to answer...…. 99 | \|___\| |
| 15.21 Are you afraid that something bad is going to happen to you? | Yes…………………………. 1  No ………………………... 0  Doesn´t know / refuses to answer …... 99 | \|___\| |
| 15.22 Do you feel happy most of the time? | No …………………………. 1  Yes…………………………... 0  Doesn´t know / refuses to answer….... 99 | \|___\| |
| 15.23 Do you often feel helpless? | Yes…………………………. 1  No ………………………... 0  Doesn´t know / refuses to answer..…. 99 | \|___\| |
| 15.24 Do you prefer to stay at home, rather than going out and doing new things? | Yes…………………………. 1  No ………………………... 0  Doesn´t know / refuses to answer ..…. 99 | \|___\| |
| 15.25 Do you feel you have more problems with memory than most? | Yes…………………………. 1  No ………………………... 0  Doesn´t know / refuses to answer ..…. 99 | \|___\| |
| 15.26 Do you think it is wonderful to be alive now? | No …………………………. 1  Yes…………………………... 0  Doesn´t know / refuses to answer .... 99 | \|___\| |
| 15.27 Do you feel worthless the way you are now? | Yes…………………………. 1  No ………………………... 0  Doesn´t know / refuses to answer …... 99 | \|___\| |
| 15.28 Do you feel full of energy? | No …………………………. 1  Yes…………………………... 0  Doesn´t know / refuses to answer…… 99 | \|___\| |
| 15.29 Do you feel that your situation is hopeless? | Yes……………….…………. 1  No ………………….……... 0  Doesn´t know / refuses to answer...…. 99 | \|___\| |
| 15.30 Do you think that most people are better off than you are? | Yes……………….…………. 1  No ………………….……... 0  Doesn´t know / refuses to answer...…. 99 | \|___\| |

PHYSICAL EXAMINATION

16. Was weight measurement performed?

Yes_____ _________kg_____gm

No_____ _________lbs

17. Was height measurement performed?

Yes_____ _________cm

No_____

18. Was waist circumference measurement performed?

Yes_____ _________cm

No____

19. Was hip circumference measurement performed?

Yes_____ _________cm

No_____

20. Was venous blood sample collected?

Yes_____ No_____

21. Was blood pressure measurement performed?

Yes_____ SBP_________ DBP _________

No___

THANK YOU FOR YOUR PARTICIPATION
